# LINC00622 transcriptionally promotes RRAGD to repress mTORC1-modulated autophagic cell death by associating with BTF3 in cutaneous melanoma

**DOI:** 10.1038/s41419-025-07828-1

**Published:** 2025-07-12

**Authors:** Can Li, Ke Wang, Lei Zhao, Jieyu Liu, Yi Jin, Chunting Zhang, Minna Xu, Min Wang, Yanjie Kuang, Jun Liu, Liang Zhou, Qian Wen

**Affiliations:** 1 Department of Toxicology, Guangdong Provincial Key Laboratory of Tropical Disease Research, School of Public Health, Southern Medical University, Guangzhou, China; 2 Institute of Molecular Immunology, School of Laboratory Medicine and Biotechnology, Southern Medical University, Guangzhou, China; 3 School of Forensic Medicine, Southern Medical University, Guangzhou, China

**Keywords:** Melanoma, Macroautophagy

## Abstract

Autophagy plays critical and complicated roles in tumors. As the central hub of nutrient signaling and cell growth, mTOR constitutes mTORC1 to be the main gateway for modulating autophagy. Yet, the regulatory mechanisms of mTORC1-regulated autophagy in tumors are not fully deciphered. Here, we report a novel long noncoding RNA, LINC00622, which modulates mTORC1-regulated autophagy in cutaneous melanoma. Functionally, LINC00622 acts as a pro-oncogenic factor to promote proliferation, colony formation, migration and invasion in melanoma while suppressing cell death. Mechanistically, LINC00622 associates with and recruits BTF3 to transcriptionally enhance RRAGD expression for activating mTORC1 and thus inhibiting autophagic cell death, which contributes to the development of cutaneous melanoma. Our findings not only demonstrated the oncogenic role of LINC00622 via RRAGD/mTORC1 axis to repress autophagic cell death in cutaneous melanoma, but also offer novel treatment targets for melanoma therapy.

## Introduction

Cutaneous melanoma (CM, here after named as melanoma) is one of the most malignant human tumors. Although melanoma accounts fewer than 5% of all cutaneous carcinoma cases, it leads to up to 75% of deaths caused by cutaneous tumors due to its rapid dissemination and heterogeneous natures [1]. Despite the advances of therapeutic strategies, advanced melanoma is tightly associated with poor prognosis and relative short survival period (6–12 months) [2]. Fully understanding the molecular mechanisms opposing stresses and maintain homeostasis in melanoma is still a big challenge and need further in-depth exploration.

Autophagy is a dynamic process in which cells utilize lysosomes to degrade their own damaged, aged, or excessive cytoplasmic proteins and organelles under the fine-tuned regulation of autophagy-related genes [3]. Autophagy is involved in all stages of carcinogenesis, including tumorigenesis, progression, metabolism and metastasis, while playing dual roles in many tumors [4, 5]. mTOR, a serine/threonine kinase, acts as a master regulator to integrate cellular metabolism by forming two complexes, mTORC1 and mTORC2, with distinct functions and structures [6]. mTORC1 regulates various stimuli and signaling networks to stimulate the synthesis of protein, lipid, and nucleotides, while inhibits catabolic progresses like lysosomal biogenesis and autophagy [7, 8]. mTORC1 is negatively regulated by tuberous sclerosis (TSC) tumor suppressor complex (TSC1/2) [9], receiving diverse arrays of signals to modulate mTORC1 activity and cell growth. Low cellular energy levels or hypoxia activate TSC1/2 to repress the activation of mTORC1 [10, 11]. Otherwise, in the presence of amino acids, Rag GTPases are the mainly activators of mTORC1. Rag GTPases can recruit mTORC1 to the surface of the lysosome and activated mTORC1 through upstream RHEB [12]. Activated mTORC1 negatively controls autophagy by inactivating ULK1 and VPS34 complexes [13].

Long non-coding RNAs (LncRNAs) belong to a class of non-coding RNAs with length exceeding 200 nucleotides and lack of protein-coding potential [14]. LncRNAs are deeply involved in pathophysiological processes including carcinogenesis, angiogenesis, or immune regulation by acting as signals, decoys, guides and scaffolds [15] to regulate diverse cellular processes in chromatin modification, transcriptional regulation, post-transcriptional regulation, and miRNA sponging [14, 16].

Many LncRNAs directly or indirectly take part in the initiation and progression of tumors by modulating autophagy [17, 18]. For example, HAGLROS associates with mTORC1 to repress autophagy and further promote the hyperproliferation and malignant progression of gastric cancer [19]. In Glioblastoma (GBM), LINC00470 from GBM derived-exosomes activates PI3K/AKT/mTOR signaling to suppress autophagy and promote glioma cell growth [20]. LncRNA CASC19 regulates AMPK-mTOR pathway to promote autophagy and contribute to radioresistance of nasopharyngeal carcinoma [21]. Thus, the deep involvement of LncRNAs in autophagy potentiates LncRNAs as the targets for cancer therapy [22–24].

In this study, LINC00622 was identified to be significantly upregulated in melanoma tumors and cell lines and functions as a pro-oncogenic factor to promote melanoma cell proliferation, colony formation, migration, and invasiveness. Mechanistically, transcriptomic sequencing after silencing of LINC00622 identified Ras related GTP binding D (RRAGD) to be the downstream target positively regulated by LINC00622, which relies on the physical association between BTF3 and LINC00622 and binds to RRAGD locus to enhance RRAGD expression and then active mTORC1 to inhibit the following autophagic cell death. Our findings demonstrate that upregulated LINC00622 acts as a key regulator to transcriptionally enhance RRAGD expression and represses mTORC1-regulated autophagy by associating with BTF3 in cutaneous melanoma.

## Results

### LINC00622 is highly upregulated in melanoma cells and tumors

**Fig. 1:**
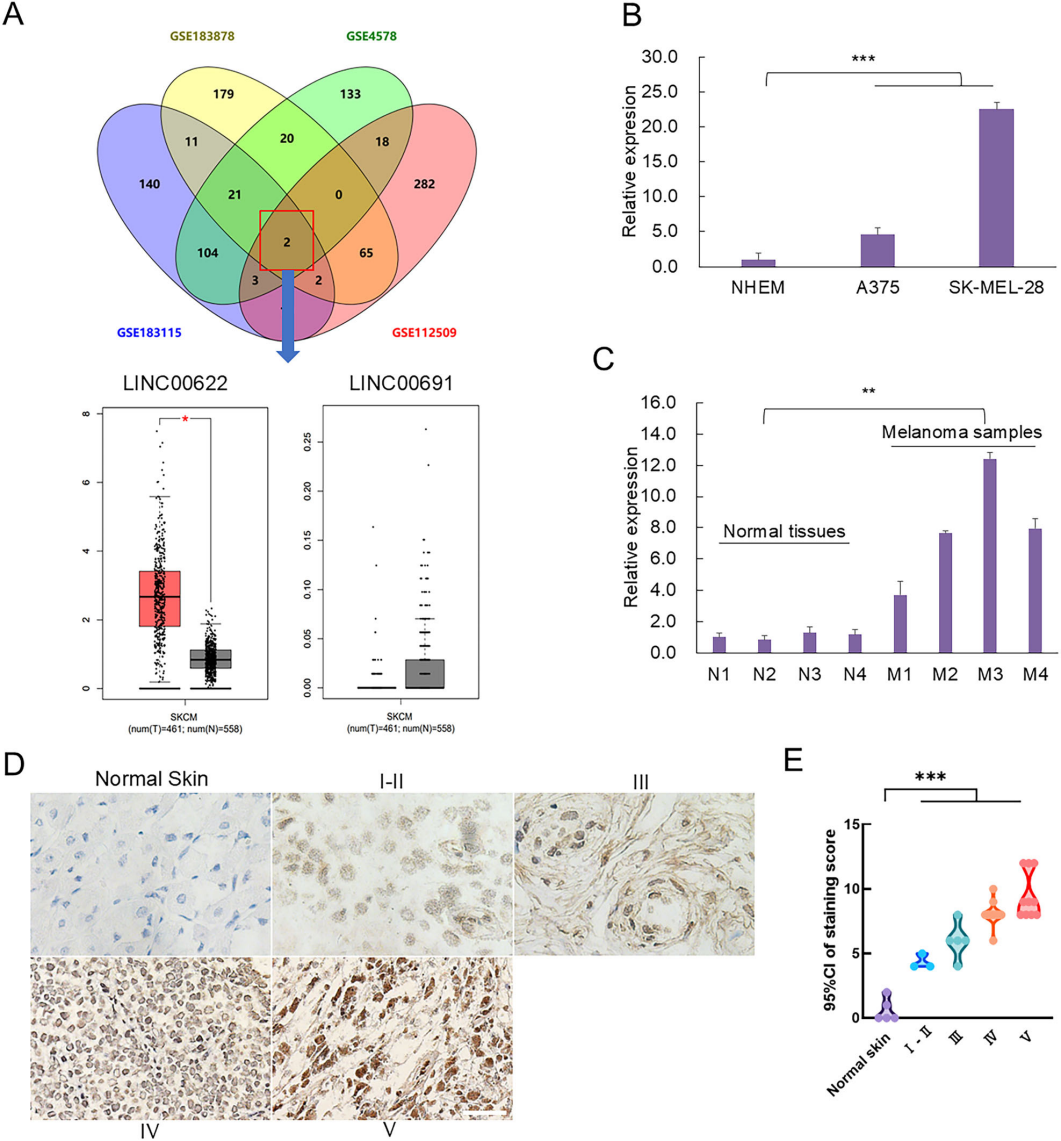
LINC00622 is upregulated in melanoma cells and tumors. **A** Screening of common significantly-upregulated LncRNAs from four public melanoma databases (GSE183878, GSE183115, GSE4587, GSE112509) and comparison of the expression levels of LINC00622 and LINC00691 between melanoma (461cases) and normal tissues (558 cases) from The Cancer Genome Atlas Program (TCGA) database. **B** The expression levels of LINC00622 were detected by qPCR in melanoma cell lines (A375 and SK-MEL-28) compared with primary normal human epidermal keratinocytes (NHEK). **C** The expression levels of LINC00622 were compared between normal cutaneous tissues and melanoma tumors. **D** ISH detection of LINC00622 on paraffin-embedded melanoma sections. Representative images were shown with various levels of staining (negative or weak in normal tissues, strong in tumor tissues). **E** The correlation of LINC00622 scoring with tumor staging [Normal skin tissues (5), I-II (3), III (5), IV (12) &V (10)]. Scale bar: 50 µm. *P < 0.05, **P < 0.01, ***P < 0.001.

To explore LncRNAs highly-expressed in melanoma, we screened four transcriptomic datasets (GSE183878, GSE183115, GSE4587, GSE112509) of melanoma including melanoma and corresponding healthy cutaneous samples. Venn analysis of the above four melanoma datasets indicated two LncRNAs, LINC00622 and LINC00691, are the common significantly-upregulated LncRNAs (Fig. 1A). We further checked such two LncRNAs expression status by interrogating the Cancer Genome Atlas Program (TCGA) database containing 461 melanoma tumor tissues and 558 normal cutaneous tissues, and confirmed LINC00622 is consistently to be significantly higher-expressed in melanoma relative to normal cutaneous tissues while LINC00691 is inconsistently lower-expressed in melanoma (Fig. 1A). Thus, LINC00622 was chosen for further analysis.

We performed qPCR to practically verify the above results and detected significantly higher expression of LINC00622 in melanoma cell lines, A375 and SK-MEL-28, compared with primary normal human epidermal melanocytes (NHEM) (Fig. 1B). To extending this analysis to clinical significance, we collected clinical specimens and demonstrated the higher expression of LINC00622 in melanoma tumors compared with normal cutaneous tissues (Fig. 1C). Specially, we performed in situ hybridization (ISH) in tissue array to detect LINC00622 expression in paraffin-embedded sections of melanoma together with normal cutaneous tissue specimens. All the melanoma samples showed strong LINC00622 signals while lower or no expression of LINC00622 could be observed in normal specimens (Fig. 1D). Further examination and scoring of LINC00622 expression indicated a positive correlation with ascending Clark stages of melanomas [Normal skin tissues (5), I-II (3), III (5), IV (12) & V (10)], a significantly increasing trend is shown across from normal cutaneous tissue and the early stages of melanoma [I-II & III) to late stages of melanoma (IV & V) (Fig. 1E). Collectively, the higher expression and tight stage-correlating expression pattern of LINC00622 in melanoma suggests LINC00622 might be deeply involved in melanoma development and potentially play critical roles.

### LINC00622 promotes cell proliferation, colony formation, migration, and invasiveness in melanoma

The expression levels of LINC00622 in different tissues and cancers are quite distinct, e.g., LINC00622 is the most highly upregulated in melanoma relative to corresponding cutaneous tissues, while it is lower-expressed in most other cancers compared with their corresponding normal tissues (Fig. S1A, B). The above results prompted that the higher expression of LINC00622 in melanoma may indicate LINC00622 probably functions as a pro-oncogenic factor and is potentially to be a candidate as one of melanoma-specific markers.

**Fig. 2:**
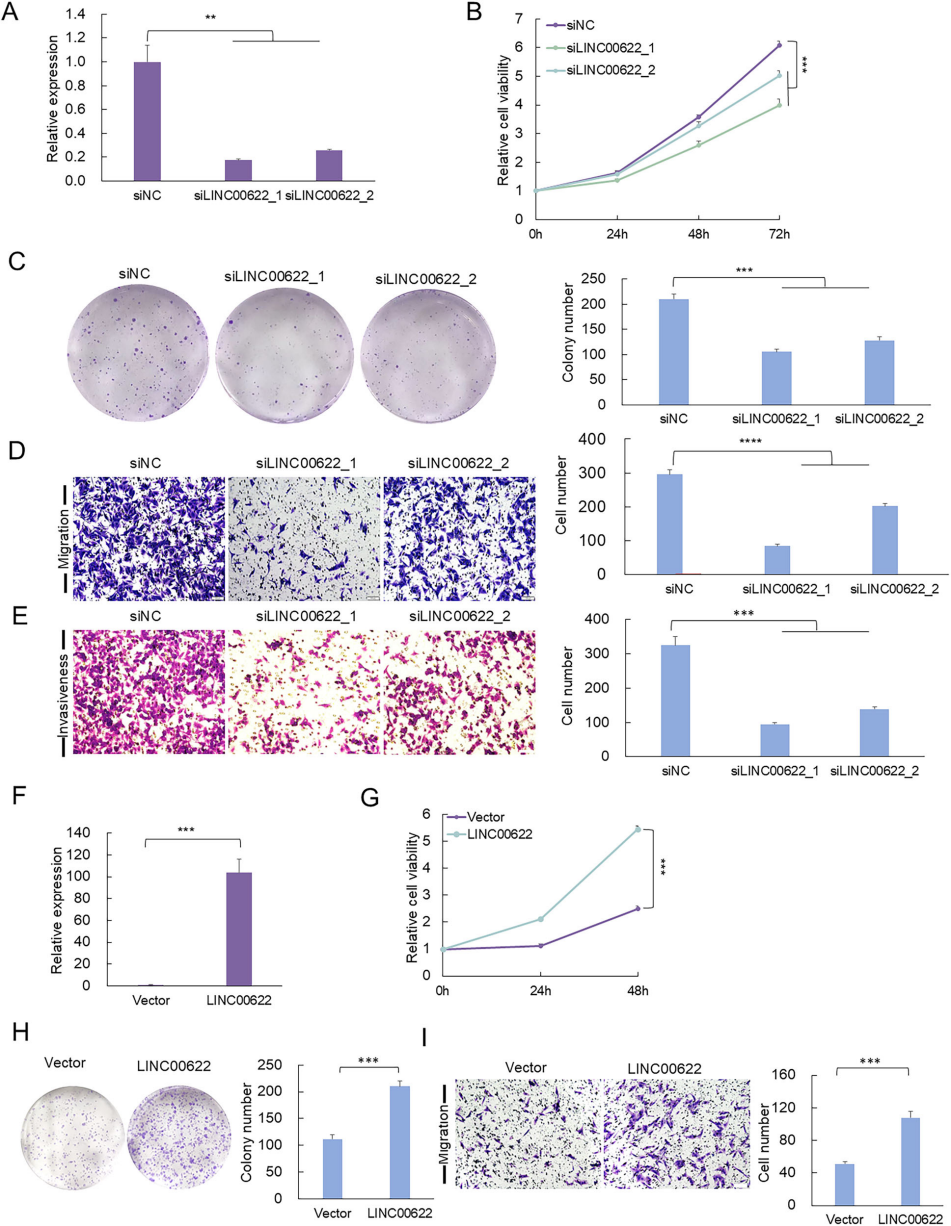
LINC00622 promotes cell proliferation, migration and invasiveness in melanoma cells. **A** LINC00622 RNA expression was detected by qPCR after knockdown of LINC00622 by RNA interference in SK-MEL-28 cells. Measurement of cell proliferation by CCK-8 assay (**B**), colony formation assay (**C**), Transwell migration assay (**D**) and Matrigel invasiveness measurement (**E**). **F** LINC00622 RNA expression was detected by qPCR after overexpression of LINC00622 in SK-MEL-28 cells. Measurements of cell proliferation by CCK-8 assay (**G**), colony formation assay (**H**) and Transwell migration assay (**I**) were performed in SK-MEL-28 cells overexpressing LINC00622. Scale bar: 100 µm. One-way ANOVA and Dunnett’s multiple comparison test. Means ± s.d. *P < 0.05, **P < 0.01, ***P < 0.001.

To test this hypothesis, we conducted RNA interference to silence LINC00622 (Figs. 2A, S2A), which led to significant decreases of proliferative capacity (Figs. 2B, S2B) and colony formation (Fig. 2C and Fig. S2C) in SK-MEL-28 and A375 cells, respectively. Further, Transwell migration and Matrigel invasiveness assays showed that depletion of LINC00622 resulted in compromised migration (Figs. 2D, S2D) and invasiveness capacities (Fig. 2E) compared with control group. Conversely, overexpression of LINC00622 in melanoma cells promoted cell proliferation, colony formation and migration capacity (Figs. 2F–I, S2E–H). Thus, the above results demonstrated LINC00622 acts as a pro-oncogenic factor in melanoma.

### LINC00622 locates in nucleus and may modulate mTORC1-regulated autophagy

**Fig. 3:**
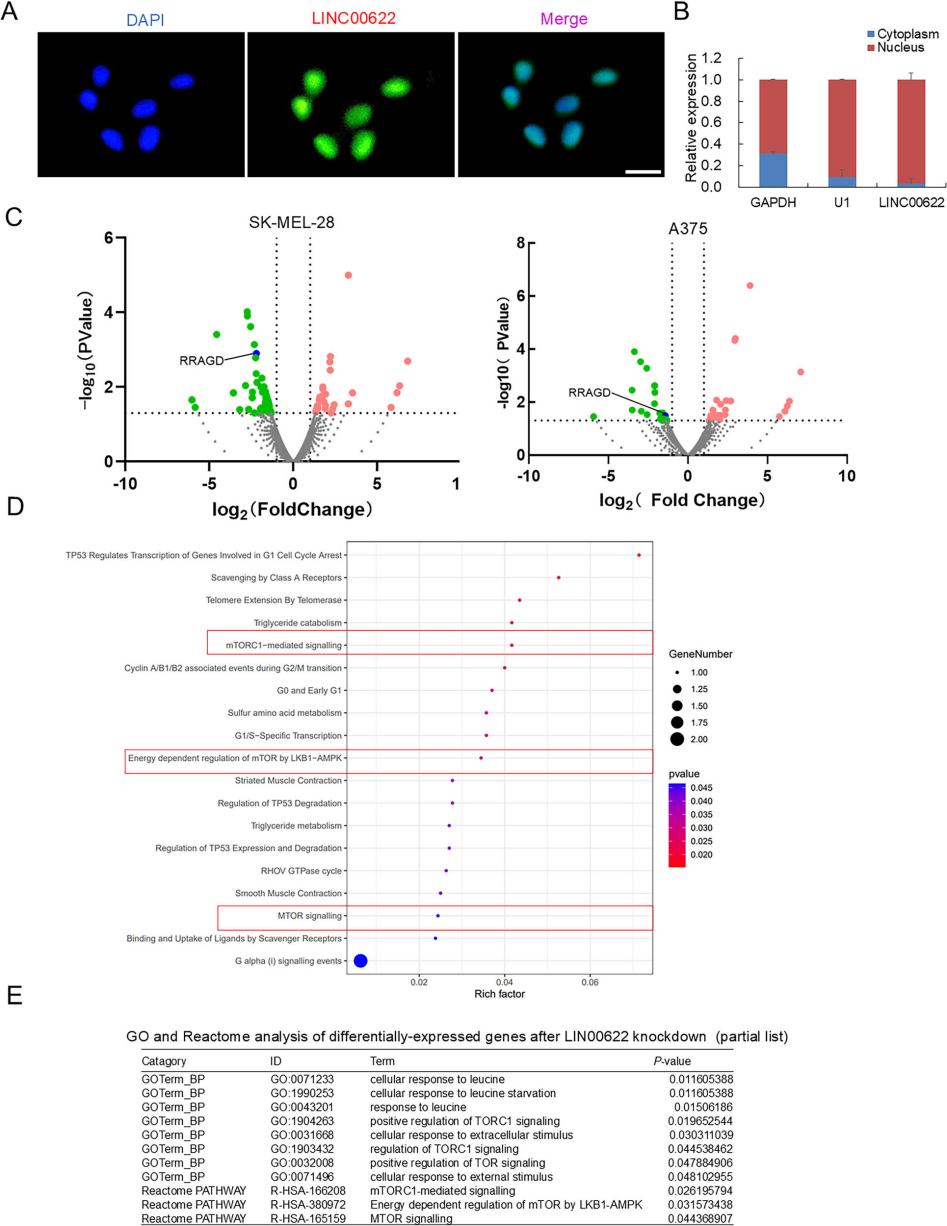
LINC00622 locates in nucleus and regulates mTORC1-related signaling. **A** Detection of the subcellular localization of LINC00622 in melanoma cells by RNA fluorescence in situ hybridization (FISH). Scale bars, 20 µm. **B** Quantitative analysis of the distributive ratio of LINC00622 in cytoplasm and nucleus in melanoma cells. **C** Differentially expressed genes from siLINC00622-treated SK-MEL-28 and A375 melanoma cells were determined by transcriptomic sequencing (RNA-Seq) and shown by volcano plots. **D** Reactome functional and signaling pathway analysis of the differentially-expressed genes after LINC00622 knockdown. **E** Gene Ontology (GO) and Reactome analysis of the differentially-expressed genes after LINC00622 knockdown. Cellular response and mTORC1 signaling-related categories are partially listed.

Different cellular localization endorses differential functions of LncRNAs. We detected the subcellular localization of LINC00622 by fluorescent in situ hybridization (FISH) and nuclear-cytoplasmic fractionation. The results indicated LINC00622 localizes mainly in nucleus (Fig. 3A, B). To probe the underlying mechanisms of oncogenic LINC00622 in melanoma, transcriptomic sequencing (RNA-Seq) was performed after loss of LINC00622. Based on the screen criteria (fold change > 1.5, *P* < 0.05), 39 upregulated and 69 downregulated genes were identified in response to LINC00622 knockdown in SK-MEL-28 melanoma cells (Fig. 3C and Supplementary Table S1), while 29 upregulated and 25 downregulated genes were identified in response to LINC00622 knockdown in A375 cells (Fig. 3C and Supplementary Table S2). By Reactome functional and signaling pathway analysis of the above differentially-expressed genes in melanoma cells, the results showed the top-ranked lists including “mTORC1-mediated signaling”, “Energy dependent regulation of mTOR by LKB1-AMPK”, “MTOR signaling” (Fig. 3D and Supplementary Table S3). Gene Ontology (GO) analysis indicated the category list consistently includes “Cellular response to external stimulus”, “response to leucine”, “cellular response to leucine”, “cellular response to leucine starvation”, “positive regulation of TORC1 signaling”, “response to starvation”, “regulation of TORC1 signaling”, “positive regulation of TOR signaling” in melanoma cells (Fig. 3E and Supplementary Table S3). We noticed most of the listed enriched categories contain RRAGD, a gene plays key roles in regulating the activation of mTORC1. Importantly, RRAGD was significantly downregulated in both SK-MEL-28 and A375 cells after silencing of LINC00622 (Fig. 3C). In short, the role of LINC00622 appears tightly related with mTORC1-regulated signaling pathways.

### LINC00622 regulates mTORC1-modulated autophagy through RRAGD

**Fig. 4:**
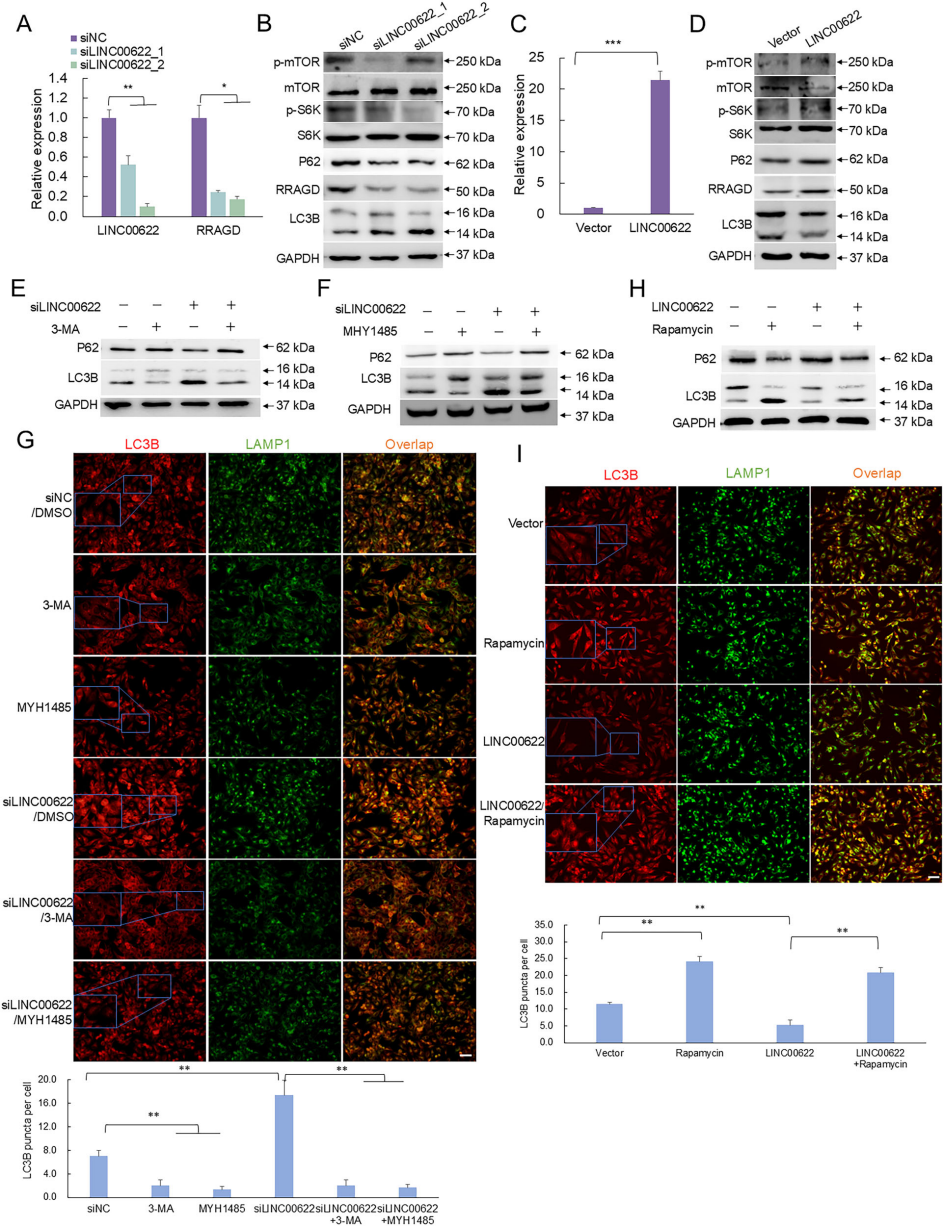
LINC00622 regulates mTORC1-modulated autophagy through RRAGD. **A** LINC00622 and RRAGD RNA expression was detected by qPCR after knockdown of LINC00622 by siRNAs in melanoma cells. **B** Western blot detection of the levels of RRAGD, p-mTOR, p-S6K, LCB-II and P62 after LINC00622 depletion. **C** qPCR detection of LINC00622 expression after LINC00622 overexpression. **D** Western blot detection of the levels of RRAGD, p-mTOR, p-S6K, LCB-II and P62 after LINC00622 overexpression. The role of LINC00622 in regulating autophagy was validated using autophagy inhibitors 3-MA and MYH1485 treatments in melanoma cells following LINC00622 silencing by Western blot detection of LC3B and P62 (**E**, **F**) and IF staining of LC3B (**G**). The role of LINC00622 in regulating autophagy was validated using autophagy activator Rapamycin following LINC00622 overexpression by Western blot detection of LC3B and P62 (**H**) and IF staining of LC3B (**I**). Scale bar, 50 µM.

From the above analysis, we suppose LINC00622 may potentially regulate mTORC1 pathway and further autophagy through the above identified RRAGD. To verify whether LINC00622 activates mTORC1 via RRAGD, we first detected RRAGD expression, phosphorylated mTOR (p-mTOR) and S6K (p-S6K) indicating the active status of mTORC1 pathway. The results showed silencing of LINC00622 downregulated RRAGD expression in both RNA and protein levels (Figs. 4A, B, S3A, B) and the amount of p-mTOR and p-S6K compared with siNC group (Figs. 4B, S3B). In contrast, overexpression of LINC00622 induced RRAGD expression and the amount of p-mTOR and p-S6K (Figs. 4C, D, S3C, D). The expression of RRAGD together with partner RRAGC were checked in melanoma tumors and cells. The results clearly indicated the mRNA and protein expression levels of RagC and RagD were significantly higher in melanoma tumors and cells compared with normal groups (Fig. S4A–F). Considering the subcellular localization of LINC00622 is in nucleus, we hypothesized that LINC00622 modulates autophagy through transcriptionally promoting RRAGD expression and further modulating mTORC1 pathway. The expression levels of LINC00622 were knocked down and the autophagy-related features were detected. Loss of LINC00622 significantly increased the amount of LC3B-II and reduced P62 levels (Figs. 4B, S3B), while the formation of LC3B foci in melanoma cells increased (Fig. S3E), indicating the enhancement of autophagic flux. At the same time, overexpression of LINC00622 decreased LC3B-II level and led to P62 accumulation (Figs. 4D, S3D) and suppressed the formation of LC3B foci in melanoma cells (Fig. S3F).

To validate whether LINC00622 regulates autophagy through mTORC1 pathway, we treated melanoma cells with autophagy inhibitor MHY1485 (a potent activator of mTORC1 to inhibit the fusion between autophagosomes and lysosomes) and 3-MA (a PI3K inhibitor widely used to inhibit autophagy). In addition to the apparent decrease of LC3B-II in autophagy inhibitor treated groups, the highly upregulated amount of LC3B-II induced after LINC00622 depletion was also repressed by autophagy inhibitor treatments (Figs. 4E, F, S3G, H). The higher LC3 foci positive cells were also reduced after autophagy inhibitor treatment (Fig. 4G). Further, we treated melanoma cells with autophagy activator Rapamycin, an allosteric inhibitor of mTORC1. After overexpression of LINC00622, the amount of LC3B-II decreased compared with empty vector group while LINC00622 overexpression-induced the downregulation of LC3B-II was inversely increased by Rapamycin treatment (Figs. 4H, S3I). Correspondingly, the Rapamycin-induced LC3 foci positive cells were repressed by LINC00622 overexpression (Fig. 4I). Collectively, LINC00622 indeed regulates RRAGD expression and negatively regulates autophagy through mTORC1 pathway.

**Fig. 5:**
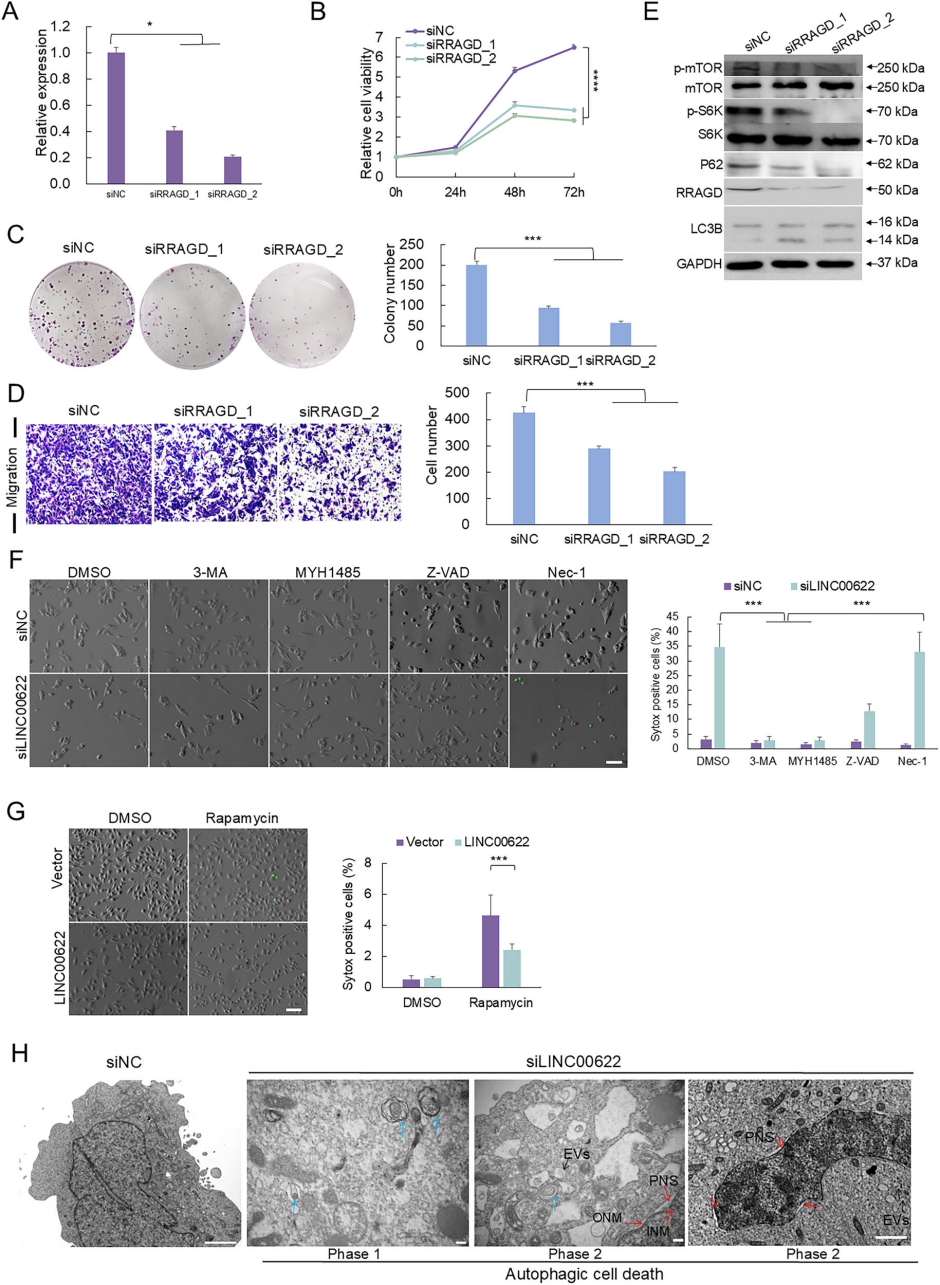
Loss of RRAGD inhibits melanoma progression and LINC00622 represses autophagic cell death. **A** RRAGD RNA expression was detected by qPCR after knockdown of RRAGD by RNA interference in SK-MEL-28 melanoma cells. Measurement of cell proliferation by CCK-8 assay (**B**), colony formation assay (**C**), Transwell migration assay (**D**) were performed. **E** RRAGD, p-mTOR, p-S6K, LC3B and P62 were detected by Western blot in SK-MEL-28 melanoma cells after RRAGD depletion. **F** Sytox green staining was performed to detect the major type of cell death induced by LINC00622 depletion in melanoma cells together with 3-MA, MYH1485, Z-VAD-FMK or Nec-1 treatments. Scale bar, 100 μM. **G** Evaluate the role of LINC00622 in repressing Rapamycin-induced cell death in melanoma cells by Sytox green staining. Scale bar, 100 μM. **H** Transmission electron microscopy (TEM) was performed to detect the type of cell death in melanoma cells. Blue arrows indicate autophagosomes. Typical features of autophagic cell death includes the formation of empty vacuoles (EVs) indicated by black arrows and perinuclear space (PNS) showing the separation of outer nuclear membrane (ONM) and inner nuclear membrane (INM) indicated by red arrows. Scale bar, 2 μM.

To further validate whether LINC00622 regulates RRAGD-mediated autophagy, knockdown of RRAGD was achieved by RNA interference (Figs. 5A, S5A), which led to significant decreases of proliferative capacity (Figs. 5B, S5B), colony formation (Fig. 5C and Fig. S5C), and migration capacity (Figs. 5D, S5D) in both of SK-MEL-28 and A375 cells. Knockdown of RRAGD induced the amount of p-mTOR and p-S6K compared with siNC group (Figs. 5E, S5E). Depletion of RRAGD significantly increased the amount of LC3B-II and reduced P62 expression (Figs. 5E, S5E) in melanoma cells, which indicates the enhancement of autophagic flux.

### LINC00622 represses autophagic cell death

The roles of autophagy could be classified as pro-survival autophagy and anti-survival autophagic cell death [25]. To make ensure the exact role of LINC00622-regulated autophagy in melanoma, we first performed Sytox Green (a nucleic dye excluded by live cells) exclusion assay and found that knockdown of LINC00622 induced significant increase of cell death rate (Fig. 5F). To discriminate which type of cell death is regulated by LINC00622, melanoma cells with LINC00622 knockdown were treated with different cell death inhibitors including 3-MA (autophagic cell death), MYH1485 (autophagic cell death), Z-VAD-FMK (apoptosis) and Nec-1 (necrosis). Nec-1 treatment almost did not influence LINC00622-induced cell death, while Z-VAD-FMK partially reduced such death rate. Interestingly, 3-MA and MYH1485 treatments fully rescued the cell death induced by LINC00622 silencing (Figs. 5F, S6A). Further, overexpression of LINC00622 could eliminate the drastic autophagic cell death induced by Rapamycin (Figs. 5G, S6B). Moreover, we performed fluorescence-activated cell sorting (FACS) analysis with Annexin V-FITC/PI double staining and found that LINC00622 depletion did not change the apoptosis rate of melanoma cells (Fig. S6C). To further verify the consequences after silencing of LINC00622, melanoma cells were observed using transmission electron microscopy. Loss of LINC00622 significantly induced the formation of autophagosomes and autolysosomes (Fig. 5H). Specially, representative features indicating autophagic cell death, like the formation of empty vacuoles (EVs) and perinuclear space (PNS) showed by the separation of outer nuclear membrane (ONM) and inner nuclear membrane (INM), are easily observed in LINC00622 knockdown group (Fig. 5H), which are the direct evidences for the presence of autophagic cell death. Meanwhile, dilation and fragmentation of ER and even loss of organelles can be observed in LINC00622-depleted cells. Such results indicated that LINC00622 plays roles mainly in protecting melanoma cells against autophagic cell death.

### LINC00622 directly associates with BTF3 and stabilizes its binding at RRAGD locus

**Fig. 6:**
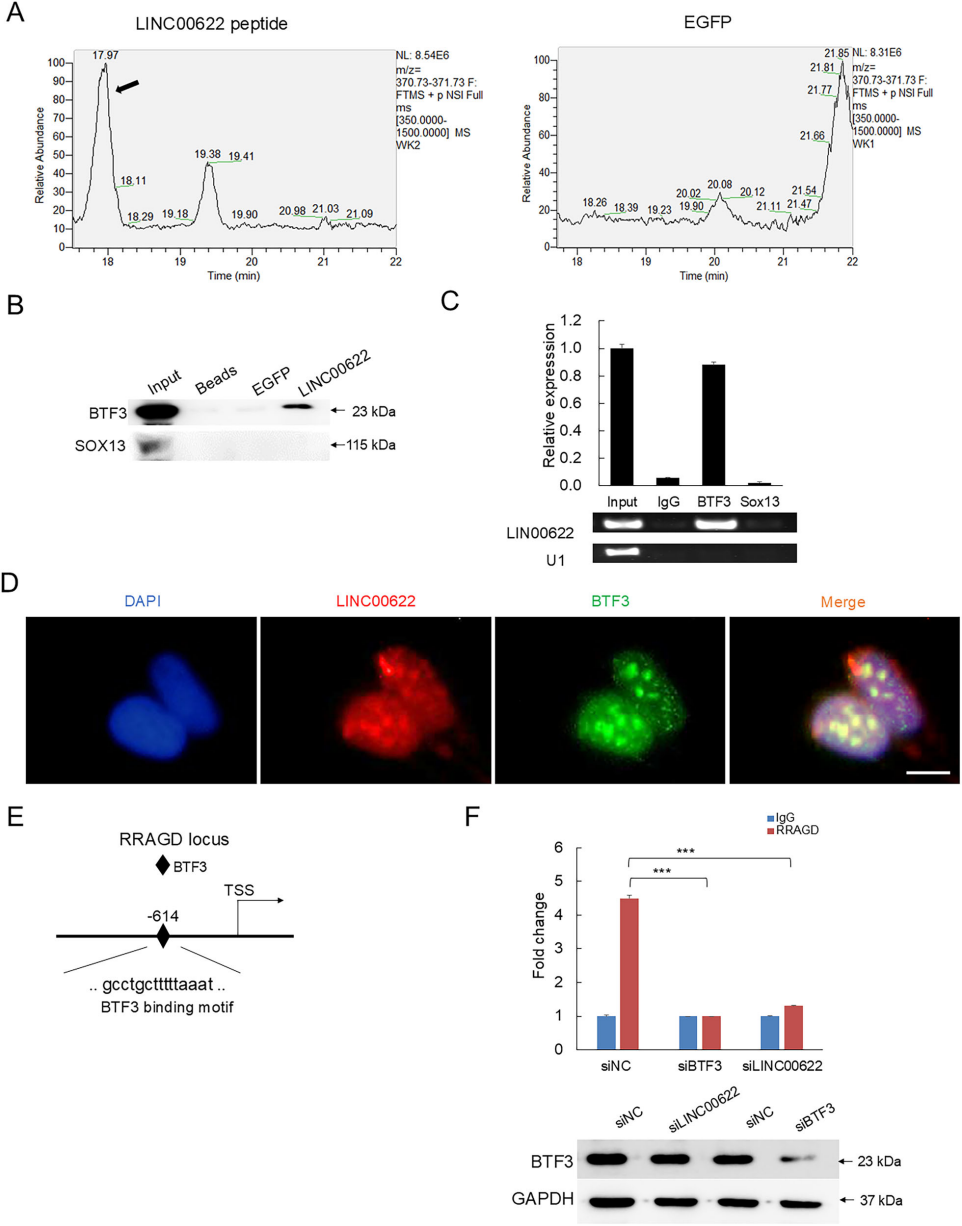
LINC00622 directly associates with BTF3 and binds to promoter region of RRAGD. **A** The chromatographic analysis of the peptide mixes pulled down by LINC00622. Arrow indicates the identified BTF3 peptide peak in the LINC00622-pulldown sample, which is lack in control EGFP sample. **B** Biotin-labeled LINC00622 transcript was used to retrieve interacting protein partners by RNA pulldown with beads only and EGFP RNA as controls in protein mix from melanoma cells. Western blot detection showed LINC00622 specific associates with BTF3 but not SOX13. **C** RNA immunoprecipitation (RIP) assay was performed using antibodies against BTF3 and SOX13 while IgG was used as control. The retrieved LINC00622 RNA was detected by qPCR. U1 transcript was used as negative control. **D** Fluorescence in situ hybridization (FISH) and immunofluorescence (IF) were performed to examine the co-localization of LINC00622 (red) and BTF3 (green) in melanoma cells. Scale bars, 20 μm. **E** Predicted binding site of BTF3 (diamond) at the promoter region of RRAGD by rVista (https://rvista.dcode.org/). **F** The binding enrichment of BTF3 at the predicted binding site on RRAGD locus was detected by ChIP-qPCR after knockdown of BTF3 or LINC00622.

LncRNAs generally execute their functions through associating with protein partners. Specially, to regulate gene transcription, LncRNAs will commonly associate with specific transcription factors or epigenetic modulators to recruit and stabilize them at specific gene loci in genome to activate or inhibit gene expression [26, 27]. Given LINC00622 mainly located in nucleus and the positively-correlated expression between RRAGD and LINC00622, we assumed a potential transcriptional regulatory mechanism may exist. RNA pulldown assay was conducted using in vitro-transcribed full-length LINC00622 RNA accompanied with a control EGFP RNA. Then, the specific binding proteins of LINC00622 were identified using high-performance liquid chromatography-mass spectrometry (HPLC-MS). There are total five transcription factors detected including CFAP20, SNF2L1, ETV6, SOX13, and BTF3, among which the gene BTF3 and SOX13 were ranked as top 2 highest-expressed genes in above transcriptomic sequencing and thus chosen for further validations. In addition to the finding of specific peptide of transcription factor BTF3 was identified by HPLC-MS (Fig. 6A and Supplementary Table S4), the specific binding between LINC00622 and BTF3 was further verified by western blot detection of BTF3 in the RNA-pulldown-precipitated protein mix (Fig. 6B). Further, RNA immunoprecipitation (RIP) also confirmed the specific interaction between LINC00622 and BTF3 (Fig. 6C), which was also supported by co-localization detection using FISH for LINC00622 and immunofluorescence (IF) staining for BTF3 respectively (Fig. 6D). In contrast, no direct interaction was detected between LINC00622 and SOX13 (Fig. 6B), highlighting the importance of experimental validation in confirming the direct association between biomolecules. Through binding site prediction as previously described [28, 29], the specific binding site of BTF3 on the genomic locus of RRAGD was predicted using rVista (https://rvista.dcode.org/) (Fig. 6E). Subsequent validations using chromatin immunoprecipitation (ChIP) demonstrated that the binding enrichment of BTF3 at RRAGD locus was significantly decreased in response to BTF3 depletion (Fig. 6F). Importantly, loss of LINC00622 significantly reduced the binding enrichment of BTF3 at RRAGD locus while did not influence BTF3 protein expression levels (Fig. 6F). Thus, the above results suggest LINC00622 determines the specific enrichment of BTF3 at RRAGD promoter region.

### The repression of RRAGD-modulated autophagy is dependent on BTF3

**Fig. 7:**
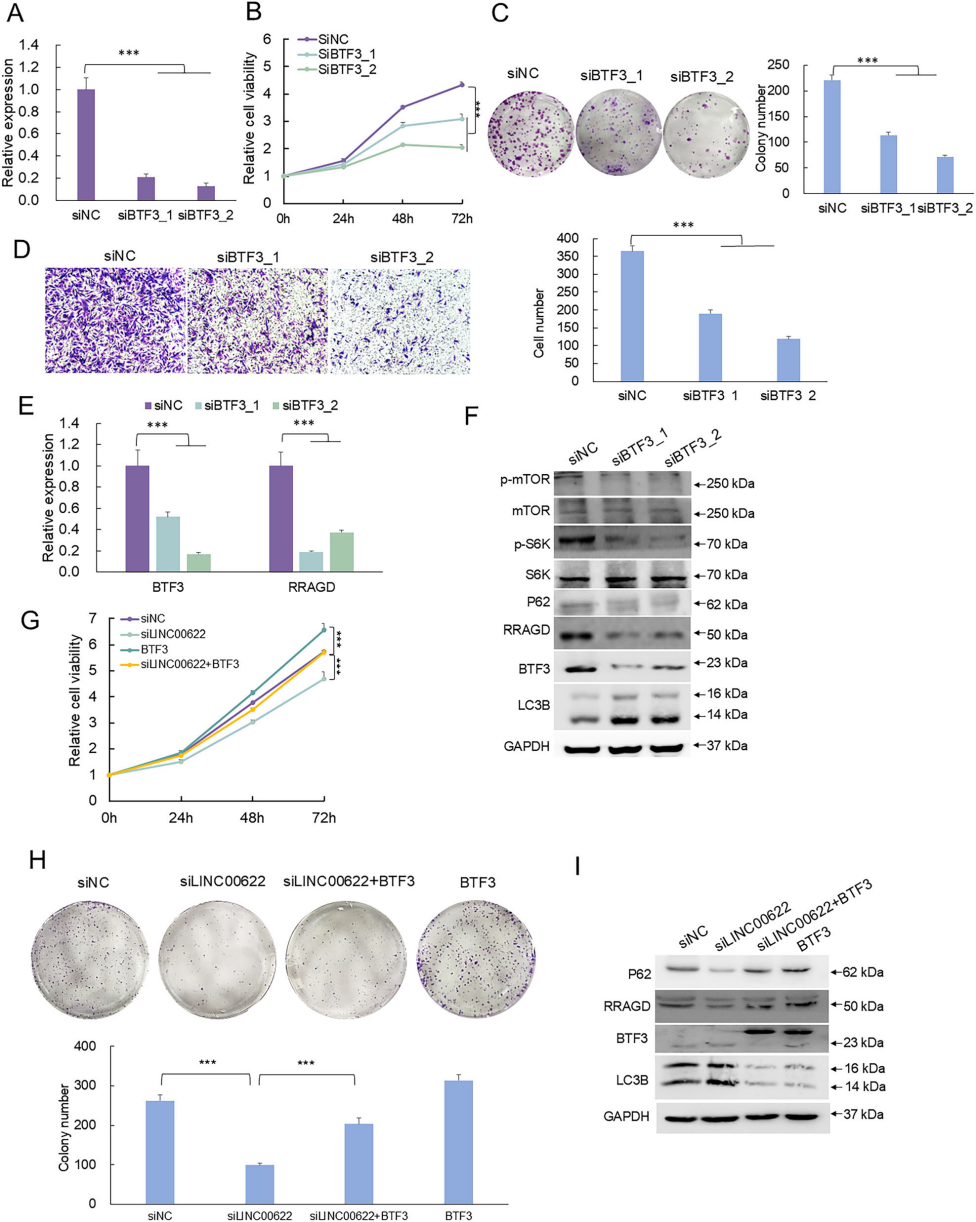
The repression of RRAGD-modulated autophagy is dependent on BTF3. **A** BTF3 RNA expression was detected by qPCR after knockdown of BTF3 by RNA interference in SK-MEL-28 melanoma cells. Measurement of cell proliferation by CCK-8 assay (**B**), colony formation assay (**C**), Transwell migration assay (**D**) were performed. (**E**) BTF3 and RRAGD were detected by qPCR after knockdown of BTF3 by RNA interference. **F** BTF3, RRAGD, p-mTOR, p-S6K, LC3B and P62 were detected by Western blot in SK-MEL-28 melanoma cells after LINC00622 depletion. Proliferative capacity (**G**) and colony formation (**H**) assays in melanoma cells in response to silencing of LINC00622 could be rescued by overexpression of BTF3. **I** The enhanced LC3-II generation together with decreased P62 and RRAGD levels after LINC00622 depletion could be significantly tuned back by BTF3 overexpression.

To examine the functions of BTF3 in the development of melanoma and RRAGD-modulated autophagy, knockdown of BTF3 was achieved by RNA interference (Figs. 7A, S7A), which led to significant decreases of proliferative capacity (Figs. 7B, S7B), colony formation (Figs. 7C, S7C), and migration capacity (Figs. 7D, S7D) in both SK-MEL-28 and A375 melanoma cells. The above results demonstrated LINC00622 may recruit BTF3 to transcriptionally regulate RRAGD, in which BTF3 is the key mediator for the execution of LINC00622 function. To determine whether BTF3 is critical for RRAGD expression and RRAGD-mediated autophagy regulated by LINC00622, we knocked down BTF3 and detected mRNA and protein expression of RRAGD (Figs. 7E, F, S7E, F). Loss of BTF3 resulted in significant downregulation of RRAGD in protein levels together with repressed levels of p-mTOR and p-S6K, enhanced generation of LC3B-II and decreased level of P62 (Figs. 7F, S7F). Such results suggested modulation of RRAGD expression and the following mTORC1 pathway by LINC00622 is rely on BTF3. At the same time, experimental results showed that the decreased proliferative capacity (Figs. 7G, S7G) and colony formation (Figs. 7H, S7H) even the downregulated RRAGD expression induced by silencing of LINC00622 could be rescued by overexpression of BTF3. Especially, the enhanced LC3-II generation together with decreased P62 and RRAGD levels after LINC00622 depletion could be significantly tuned back by BTF3 overexpression (Figs. 7I, S7I). The above results suggest that BTF3 is the key partner for LINC00622 to establish the transcriptional regulation of RRAGD and the following inhibition of autophagic cell death in melanoma.

### Loss of LINC00622 compromises melanoma growth by enhancing autophagic cell death in vivo

**Fig. 8:**
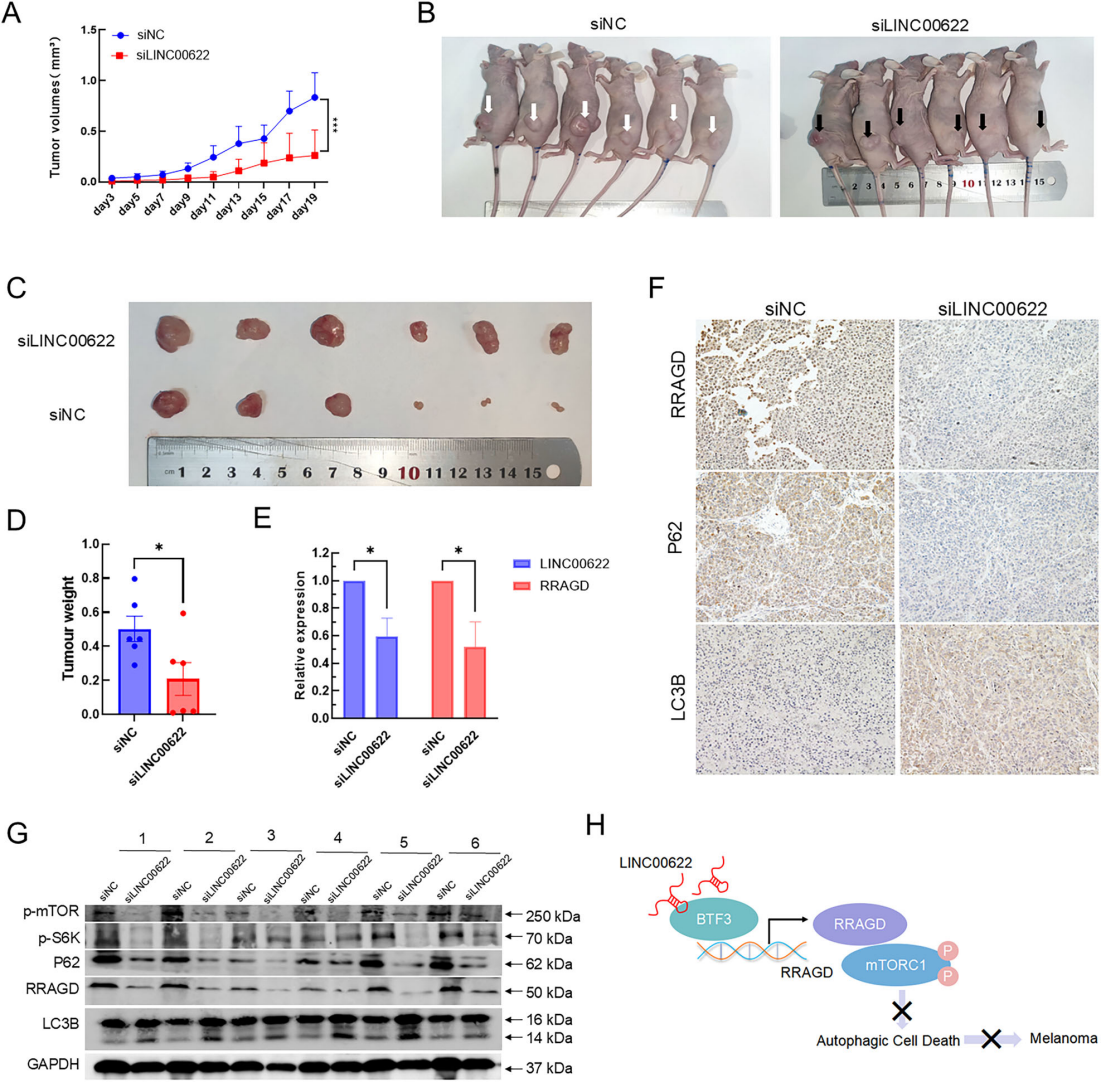
Loss of LINC00622 compromises melanoma growth by enhancing autophagic cell death in vivo. **A** Loss of LINC00622 inhibited melanoma growth in a mouse xenograft model. Tumor volumes (mm^3^) were plotted according to day. **B**, **C** The mice were sacrificed at the end of the experiment and the dissected xenografts were shown. Black and white arrows respectively indicate the siNC-treated and siLINC00622-treated xenografts. **D** The weight comparison of xenografts between siNC-treated and siLINC00622-treated groups. **E** The expression of LINC00622, RRGAD was detected in dissected xenografts by qPCR. Statistical data of qPCR represented the average of four independent experiments ± SE. **F** The expression levels of LC3B, P62, and RRAGD in tumor sections were evaluated using IHC staining. Scale bar, 50 µm. **G** The protein levels of p-mTOR, p-S6K, LC3B, P62, and RRAGD were detected in xenografts after LINC00622 knockdown by Western blot. **H** A model depicts that LINC00622 transcriptionally enhances RRAGD expression to repress autophagic cell death by associating with BTF3 to promote cutaneous melanoma progression.

To evaluate the pro-carcinogenic and anti-autophagic functions of LINC00622 in vivo, we established a xenograft tumor model in immunocompromised mice. Initially, no significant difference of tumor sizes could be observed between siNC control group and siLINC00622-treated group. After 7 days, depletion of LINC00622 led to markedly-compromised size of xenografts (Fig. 8A) and tumor mass formation is apparent smaller compared with siNC group (Fig. 8B–D). The knockdown efficiency of LINC00622 expression in siLINC00622-treated group was verified by qPCR in xenograft tumors (Fig. 8E) and accompanied with significantly-decreased expression of RRAGD. Furthermore, LINC00622 depletion led to significant upregulation of LC3B-II together with repression of RRAGD, P62, p-S6K, and p-mTOR (Fig. 8F, G). Collectively, the in vivo experiments indicated that LINC00622 suppresses autophagy by repressing RRAGD expression and further inhibiting mTORC1-mediated autophagic cell death to promote melanoma progression.

## Discussion

Programmed cell death (PCD) is a special type of cellular response to a variety of cellular stresses and/or extrinsic stimuli that control cell fate in metazoans [30]. Induction of PCD in tumors is the critical step during cancer treatments [31], while suppression of PCD is one of the key mechanisms for tumor survival and development. Autophagy fine-tuned by autophagy-associated genes plays crucial roles in tumor development and may inhibit or promote tumor development [32]. Notably, persistent and uncontrolled autophagic flux can lead to autophagic cell death, which is characterized by the presence of typical empty vacuoles, loss of organelles, ER fragmentation, mitochondrial electron densification, focal plasma membrane disruption, and nuclear concavity [31, 33].

Autophagic cell death belongs to type II programmed cell death. Considering the autophagy origin, autophagic cell death could be completely inhibited by blocking of autophagy pathway [30]. In this study, silence of LINC00622 resulted in featured autophagic cell death in melanoma cells, which can be almost fully inhibited by autophagy inhibitors (3-MA and MYH1485). Previously, we have shown LncRNA PURPL directly interacts with ULK1 to promote mTOR-mediated phosphorylation of ULK1 at Ser757 to repress autophagic cell death and promote melanoma cell survival. Depletion of PURPL leads to AMPK-mediated phosphorylation of ULK1 at Ser555 and Ser317, which promotes autophagic cell death and thereby inhibits melanoma development [25]. In this study, LINC00622 regulates autophagy through modulating the expression of RRAGD, a crucial activator for mTORC1, to suppress mTORC1-regulated autophagy. In the presence of LINC00622, mTORC1 is recruited to lysosomes by RRAGD and activated by GTPase RHEB to subsequently inhibit autophagy. Silencing of LINC00622 reduced RRAGD expression, leading to mTORC1 inhibition and the following activation of autophagy. Interestingly, the autophagic response regulated by LINC00622 is autophagic cell death, which compromises the survivability of melanoma and is promising in future melanoma treatment.

MTOR is the central hub regulator for tumor growth and can be abnormally activated by a variety of oncogenic factors or other epigenetic modifications. Rapamycin and Rapalogs as mTOR inhibitors have been approved to be used as single agent for treating several tumors [34, 35]. Interestingly, inducing autophagic cell death by targeting mTOR has been become a hot strategy in cancer therapy. For example, 3-bromo-N’-(4-hydroxybenzylidene)-4-methylbenzohydrazide derivatives as mTOR inhibitor to induce autophagic cell death and apoptosis in triple-negative breast cancer [36]. Apigenin has been discovered to stimulate AMPK–ULK1 activation while inhibiting mTOR, leading to autophagic cell death in gastric cancer [37]. Yet, mTORC1-modulated autophagy is less studied in melanoma. In this study, we screened out LINC00622 as one of the top-expressed LncRNAs and plays pro-carcinogenic and anti-autophagic roles in melanoma. Mechanistic study indicated that LINC00622 transcriptionally enhances RRAGD to repress mTORC1-modulated autophagic cell death to promote melanoma development.

RRAGD belongs to Ras-related GTP-binding protein family (Rags), which contains four members including RRAGA, RRAGB, RRAGC, and RRAGD. Rags act as molecular switches by binding GTP or GDP in numerous cell processes and signaling pathways [24]. Specially, Rags are considered the dominant activators of mTORC1 signaling [22, 38]. In the presence of amino acids, Rag GTPases become GTP-loaded and induce mTORC1translocation from the cytoplasm to lysosomes, and then mTORC1 is activated by RHEB [38]. Specifically, RRAGD has been reported to be one of several genes differentially expressed in human melanoma cell lines and considered as a promising target for diagnostic and therapeutic investigations [23]. circEXOC6B associates with RRAGB to antagonize HIF1A-RRAGB-mTORC1 positive feedback loop for enhancing 5-fluorouracil-induced apoptosis and repressing colorectal cancer growth [39]. In this study, evidences showed LINC00622 directly associates with BTF3 to transcriptionally regulate RRAGD expression, leading to the suppression of mTORC1-modulated autophagic cell death.

BTF3 is deeply involved in transcriptional regulation during the eukaryote growth and tumors. For example, BTF3 acts as an oncogenic transcription factor through transcriptional upregulation of Replication Factor C to promote prostate cancer [40]. BTF3 also regulates proto-oncogene BMI1 to enhance epithelial-mesenchymal transition and stem cell-like traits to promote colorectal cancer development [41]. In this study, we found the pro-oncogenic and autophagy-suppressive roles of LINC00622 are fully dependent on its interacting partner BTF3 which conducts the transcription regulation of RRAGD expression and further repression of autophagic cell death.

The study of LINC00622 in tumor is rare. In neuroblastoma, adipose-derived stem cell-derived extracellular vesicles containing LINC00622 could be absorbed by neuroblastoma cells. LINC00622 inhibits the activity of transcription factor AR and promotes GABBR1 expression to repress neuroblastoma cell growth [42]. LINC00622 has been also shown to be upregulated during fibrosis and might function in inflammatory response [43]. In our study, LINC00622 is also consistently located in nucleus and regulates the activity of transcription factor BTF3 with distinct function, which adds novel knowledge to LINC00622.

Collectively, our study identified LINC00622 to be a highly-expressed and pro-oncogenic factor to promote the proliferation, colony formation, migration and invasiveness while repressing autophagic cell death in melanoma. LINC00622 physically associates with BTF3 and binds to the RRAGD locus to transcriptionally enhance RRAGD expressions and further activate mTORC1 to inhibit autophagic cell death, which contributes to melanoma progression. Our findings highlight the oncogenic and anti-autophagic roles of LINC00622 in melanoma, which suggests a series of novel intervention targets for melanoma therapy.

## Materials and methods

### Patient samples

Commercially available human melanoma tissue array (ZL-MEL962) was obtained from Shanghai Wellbio Technology Co., Ltd., which contains 30 specimens of melanoma and 5 specimens from normal skin. The use of the above specimens in this study was approved by the Institutional Review Board of Shanghai Wellbio Technology Co., Ltd. (Shanghai, China). Fresh samples obtained during surgery were immediately frozen in liquid nitrogen, and total RNA was subsequently extracted or simultaneously paraffin-embedded. Tumors were classified according to the American Joint Committee on Cancer classification of melanoma (eighth edition) [44]. This study was approved by the Institutional Review Board of Nanfang Hospital affiliated to Southern Medical University. All patients provided written informed consent for the use of surgical samples. None of the patients had been pretreated with radiotherapy or chemotherapy before surgical excision. All investigations complied with the principles of the Declaration of Helsinki.

### Animals

Male athymic nude mice (BALB/C-nu/nu, 4–5 weeks old) purchased from the animal center of Southern Medical University were used for xenograft studies. Euthanasia of cervical dislocations was performed in order to spare mice from suffering. This study was approved by the Institutional Animal Care and Use Committee (IACUC) of Southern Medical University (Approval code L2019178) and in accordance with the guidelines of the Asian Federation of Laboratory Animal Science Associations (AFLAS) and the National Regulations for the Administration of Affairs Concerning Experimental Animals (8 January 2011). Mouse transportation, housing, and breeding were conducted according to the recommendations of “The use of non-human animals in research”.

### Cell lines

Melanoma lines A375 (Female, Wuhan Sunncell Biotechnology Co., Ltd.) and SK-MEL-28 (Male, Wuhan Sunncell Biotechnology Co., Ltd.), Human Epidermal Melanocytes (Cell Systems) were grown in Dulbecco’s modified Eagle medium (DMEM, Life Technologies) supplemented with 10% fetal bovine serum (ExCell Bio, FSP500) and maintained at 37 °C with 5% CO_2_ in a humidified atmosphere. All cell lines used in this study were verified by STR profile authentication. All cells have been tested and demonstrated to be free of mycoplasma contamination.

### RNA isolation and qPCR

Total RNA was extracted from cells or tissues by Trizol reagent (TransGen Biotech Co., Ltd.) according to the manufacturer’s instructions. Subsequently, RNA was reversely transcribed into cDNA using TransScript Uni All-in-One First-Strand cDNA Synthesis SuperMix for qPCR (One-Step gDNA Removal) (TransGen Biotech, AU341). mRNA expression analysis was performed using PerfectStart Green qPCR SuperMix (TransGen Biotech, F170) on LightCycler 96 Detection System (Roche, Basel, Switzerland). Detection of GAPDH mRNA expression was used for normalize. Primers used in this study are listed in Table S5.

### DNA constructs

The LINC00622 and BTF3 expressing constructs were purchased from YouBio Biological Technology Co., Ltd. (http://www.youbio.cn) with sequencing verifications.

### Immunoblotting and IHC assays

Extraction of animal and cellular proteins was done according to previous methods [45]. The following primary antibodies and dilutions were used: RRAGD (Cell Signaling Technology, 4470, 1:2000), Phospho-p70 (S6K) (Thr389) (Proteintech, 823-1-RR, 1:2000), Phospho-mTOR (Ser2448) (Proteintech, 80596-1-RR, 1:2000), LC3B (PTMBIO, PTM-6384, 1:4000), P62/SQSTM1 (PTMBIO, PTM-6234,1:4000), mTOR (Santa Cruz Biotechnology, sc-517464, 1:3000), S6K/p70 S6 kinase (Santa Cruz Biotechnology, sc-230, 1:3000), BTF3 (Santa Cruz Biotechnology, sc-166094, 1:3000) and GAPDH (Santa Cruz Biotechnology, sc-25778, 1:5000). The following secondary antibodies were also used: anti-mouse IgG-horseradish peroxidase (HRP), anti-rabbit IgG-HRP, and anti-goat IgG-HRP (Santa Cruz Biotechnology). Bound antibodies were visualized using Luminata Forte Western HRP substrate (Millipore). Xenograft tumors were formalin-fixed, paraffin-embedded, and sectioned for IHC staining. The following antibodies were used: RRAGD (Cell Signaling Technology, 4470,1:100), LC3B (PTMBIO, PTM-6384 1:100), P62/SQSTM1 (PTMBIO, PTM-6234,1:100).

### Transmission electron microscopy

Firstly, the cells were fixed with 2.5% glutaraldehyde and then re-fixed with 1% osmium tetroxide. The fixed samples were dehydrated with a serial concentrations of ethanol solutions (50%, 70%, 80%, 95%). The samples were incubated with the mixed solution of acetone and embedding agent for 2 h and then transferred into pure embedding agent. Finally, the embedded samples were ultra-thinly sectioned and observed with transmission electron microscope Hitachi H-7500.

### RNA immunoprecipitation assay

RIP was performed as described [27]. In short, 8 μg antibodies against BTF3 or isotype IgG (Merck Millipore) as a negative control were used to perform RIP assay.

### RNA pulldown

RNA pulldown was performed as previously described [27]. Biotinylated LINC00622 transcript and EGFP RNAs were transcribed using a MAXIscript T7/T3 in vitro transcription kit (Ambion) and Biotin RNA labeling Mix (Roche).

### High-performance liquid chromatography-mass spectrometry analysis

20 μg for each of immunoprecipitated protein mix sample was separated by sodium dodecyl sulfate-polyacrylamide gel electrophoresis (SDS-PAGE) and stained with Coomassie Brilliant Blue R250 and then processed with the Trypsin Profile IGD Kit (Sigma, PP0100). The resulting digest was treated with ZipTip C18 (Merck Millipore, ZTC18S096) then subjected to analysis by Thermo Fisher Scientific orbitrap fusion LC-MS/MS in positive ion, linear, delayed-extraction mode. Calibration was carried out using a standard peptide mixture. The mass spectra were subjected to sequence database for searching with Proteome Discoverer v2.1 software (Thermo Scientific), and the results were analyzed by Xcalibur 2.0.

### Xenograft mouse model

Briefly, xenograft mouse model 4–5-week-old female athymic nude mice were purchased from Guangdong Medical Laboratory Animal Center. Sample size was estimated as described [46]. Equal numbers of mice were randomly assigned to siNC group and siLINC00622 group (6 mice/group), respectively. 0.2 mL of the melanoma cell suspension containing 5 × 10^6^ cells was injected subcutaneously into nude mice. Tumor diameters were measured and recorded every 2 days to generate tumor growth curves. Tumor volume was determined by measuring the length (l) and width (w) of the tumor and calculating the tumor volume (V) according to the formula: *V* = lw^2^/2. After 3-week assessment, the tumors were excised and frozen to extract RNA and proteins, or paraffin-embedded for IHC evaluations.

### Cell proliferation and colony forming assays

A375 and SK-MEL-28 melanoma cells (3000 per well) cultivated on 96-well plates were transfected with siRNA, and cell proliferation was detected after 0, 24, 48, and 72 h using cell counting kit (TransGen Biotech, FP101) at 450 nm as described in the manual. For the colony forming assay, transfected cells were incubated in six-well plates at 1000 cells per well, which were maintained in DMEM with 10% FBS. At day 7, plates were collected after being washed once with PBS and fixed in 4% paraformaldehyde for 30 min, then washed with PBS again. Finally, 0.1% crystal violet was used to stain the cells. Visible colonies were photographed and counted.

### Transwell assay

To assess cell migration, 5.0 × 10^5^ A375 cells and 1.0 × 10^6^ SK-MEL-28 cells transfected with siNC or siLINC00622 were seeded onto the upper chambers of 12-well plates (Merk Millipore) in serum-free DMEM. During culture at 37 °C for 48 h, the cells in the upper chambers were attracted and migrated through 8 μM pores at the bottom of upper chamber by chemoattractant. The chambers were washed twice with PBS, fixed with 3.7% formaldehyde, permeabilized using 100% methanol at room temperature, stained with 0.1% crystalline violet, and migrated cells were observed using a microscope after remove the cells remaining in the upper chambers.

### Matrigel invasiveness assay

For the assessment of invasive ability, SK-MEL-28 cells transfected with siNC or siLINC00622 were concentrated to 1 × 10^6^ cells in cell suspension and then added to the upper chamber (Merck Millipore) coated with Matrigel for the invasion assay. Other treatments were performed as in migration assay.

### Treatments with inhibitors

Autophagy inhibitor 3-Methyladenine (3-MA) (5 mM, Selleck) and caspase inhibitor Z-VAD-FMK (100 μM, Selleck) treatments were performed in melanoma cells for 5 h after LINC00622 knockdown or overexpression. MYH1485 (100 μM, Selleck), Rapamycin (20 nM, Selleck) and Necrostatin-1 (Nec-1, 50 μM, Selleck) was treated for 24 h.

### RNA-seq data analysis

Total RNA was isolated from A375 and SK-MEL-28 cells using the Trizol Reagent (Ambion, USA) according to the manufacturer’s protocol. Briefly, the mRNA was enriched by oligodT conjugated NEBNext® Poly(A) mRNA Magnetic Isolation Module (NEB, USA) according to instructions. And then fragmented to be approximately 200 bp. Subsequently, the RNA fragments were subjected to first strand and second strand cDNA synthesis following by adaptor ligation and enrichment with a low-cycle according to instructions of NEBNext® Ultra™ RNA Library Prep Kit for Illumina. The purified library products were evaluated using Agilent 2200 TapeStation and Qubit (Thermo Fisher Scientific, USA). Libraries were sequenced using Illumina (Illumina, USA) with paired-end 150 bp at Ribobio Co. Ltd (Ribobio, China).

### Statistical analysis

Statistical tests were performed for independent samples with unpaired *t*-test or one-way ANOVA tests (SPSS version 17.0, SPSS Inc.). All statistical tests incorporated two-tailed tests and homogeneity of variance tests and were considered to reflect significant differences if *P < 0.05, **P < 0.01, or ***P < 0.001. Details of statistical analyses including sample numbers (n) are included in respective figure legends.

## Supplementary information

Supplementary Data

Supplementary Original Data

Supplementary Table S1

Supplementary Table S2

Supplementary Table S3

Supplementary Table S4

Supplementary Table S5

## Data Availability

The mass spectrometry proteomics data have been deposited to the ProteomeXchange (http://www.proteomexchange.org) Consortium via the PRIDE [47] partner repository with the dataset identifier PXD055040. RNA-seq data have been deposited in NCBI GEO datasets (accession: GSE275315). The original data of Western blots are all shown in Supplementary Original Data. All data generated or analyzed during this study are included in this published article and its Supplementary files and available from the corresponding authors on request.
