# Supplementary material for: LINC00622 transcriptionally promotes RRAGD to repress mTORC1-modulated autophagic cell death by associating with BTF3 in cutaneous melanoma
Source: Cell Death Dis. 2025 Jul 12;16(1):515. doi: 10.1038/s41419-025-07828-1 (PMC12255717; doi:10.1038/s41419-025-07828-1)
Supplement: Supplementary file 1 — Supplementary Data [file 41419_2025_7828_MOESM1_ESM.pdf]

Figure S1

A

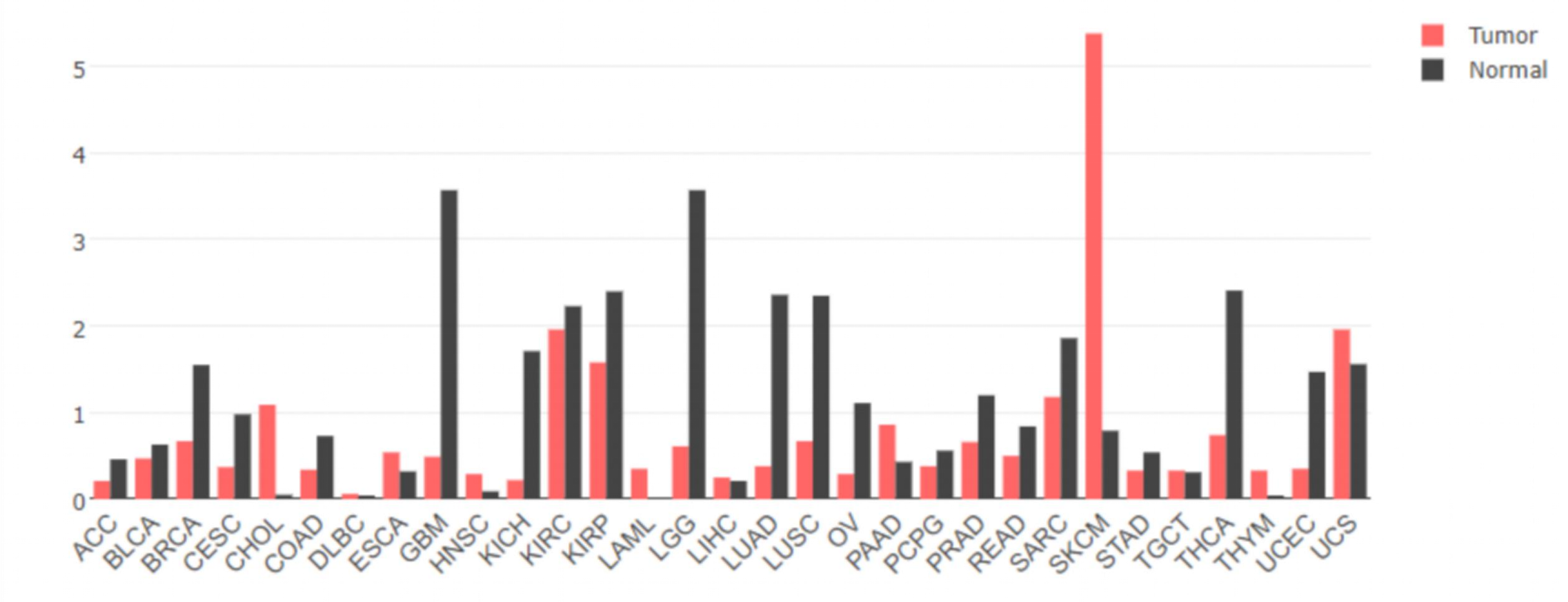

B

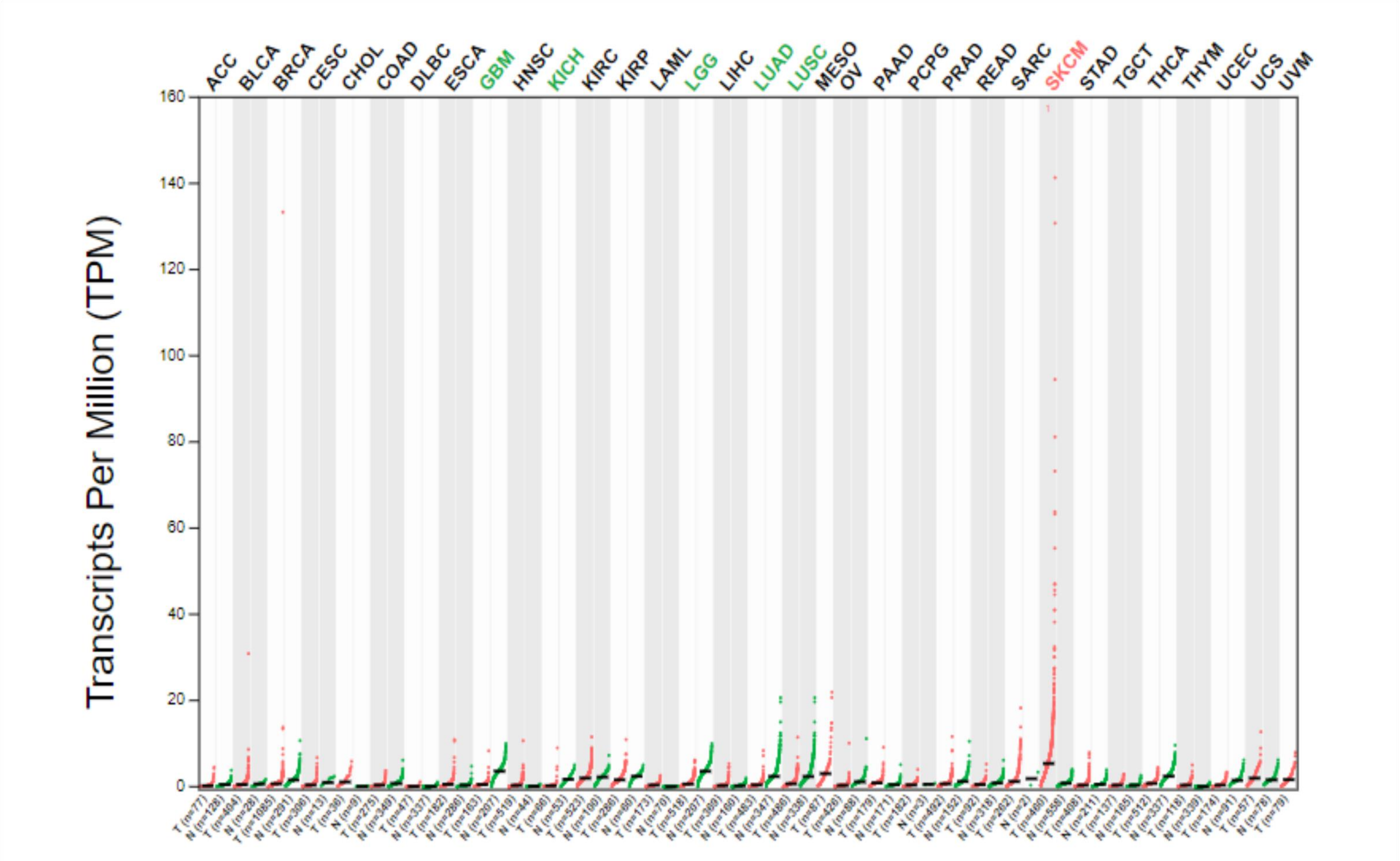

Figure S1. Normalized LINC00622 expression levels in a variety of tumors analyzed in TCGA database. (**A, B**) The normalized expression data is from The Cancer Genome Atlas Program (TCGA) database and the diagram showing the expression levels of LINC00622 in different types of cancer analyzed by Gene Expression Profiling Interactive Analysis (GEPIA, http://gepia.cancer-pku.cn/)

Figure S2

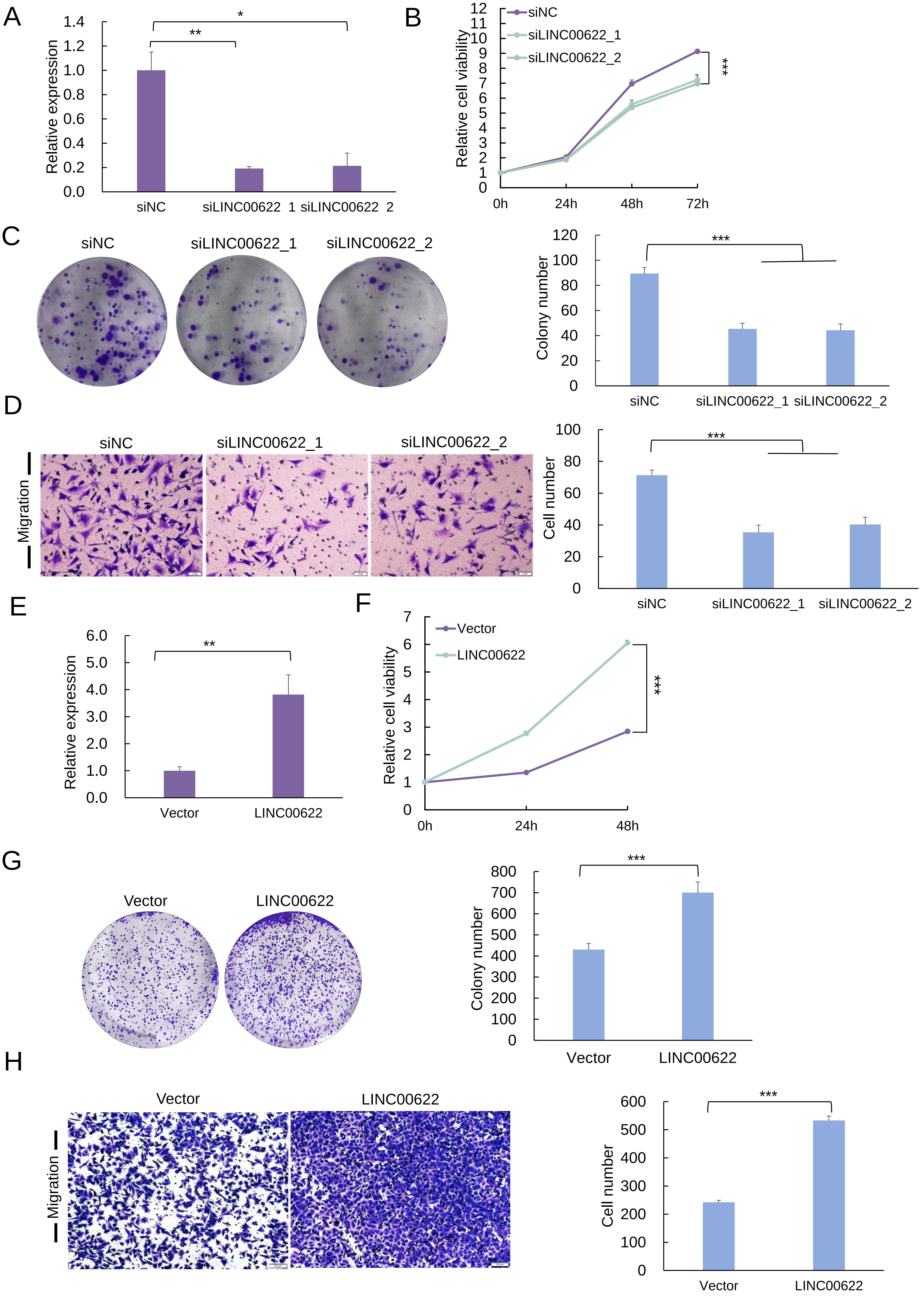
A
Relative expression
1.4
1.2
1.0
0.8
0.6
0.4
0.2
0.0
**
*
siNC
siLINC00622_1
siLINC00622_2
B
Relative cell viability
siNC
siLINC00622_1
siLINC00622_2
***
0h
24h
48h
72h
C
siNC
siLINC00622_1
siLINC00622_2
Colony number
***
siNC
siLINC00622_1
siLINC00622_2
D
siNC
siLINC00622_1
siLINC00622_2
Migration
Cell number
***
siNC
siLINC00622_1
siLINC00622_2
E
Relative expression
6.0
5.0
4.0
3.0
2.0
1.0
0.0
**
Vector
LINC00622
F
Relative cell viability
Vector
LINC00622
***
0h
24h
48h
G
Vector
LINC00622
Colony number
***
Vector
LINC00622
H
Vector
LINC00622
Migration
Cell number
***
Vector
LINC00622

Figure S2. LINC00622 promotes cell proliferation, migration and invasiveness in A375 melanoma cells. (**A**) LINC00622 RNA expression was detected by qPCR after knockdown of LINC00622 in A375 cells. Measurement of cell proliferation by CCK-8 assay (**B**), colony formation assay (**C**) and Transwell migration assay (**D**) were performed after LINC00622 depletion. (**E**) LINC00622 RNA expression was detected by qPCR after overexpression of LINC00622 in A375 cells. Measurements of cell proliferation by CCK-8 assay (**F**), colony formation assay (**G**) and Transwell migration assay (**H**) were performed in A375 cells overexpressing LINC00622. Scale bar: 100 μm. One-way ANOVA and Dunnett's multiple comparison test. Means ± s.d. *$P < 0.05$, **$P < 0.01$, ***$P < 0.001$.

Figure S3

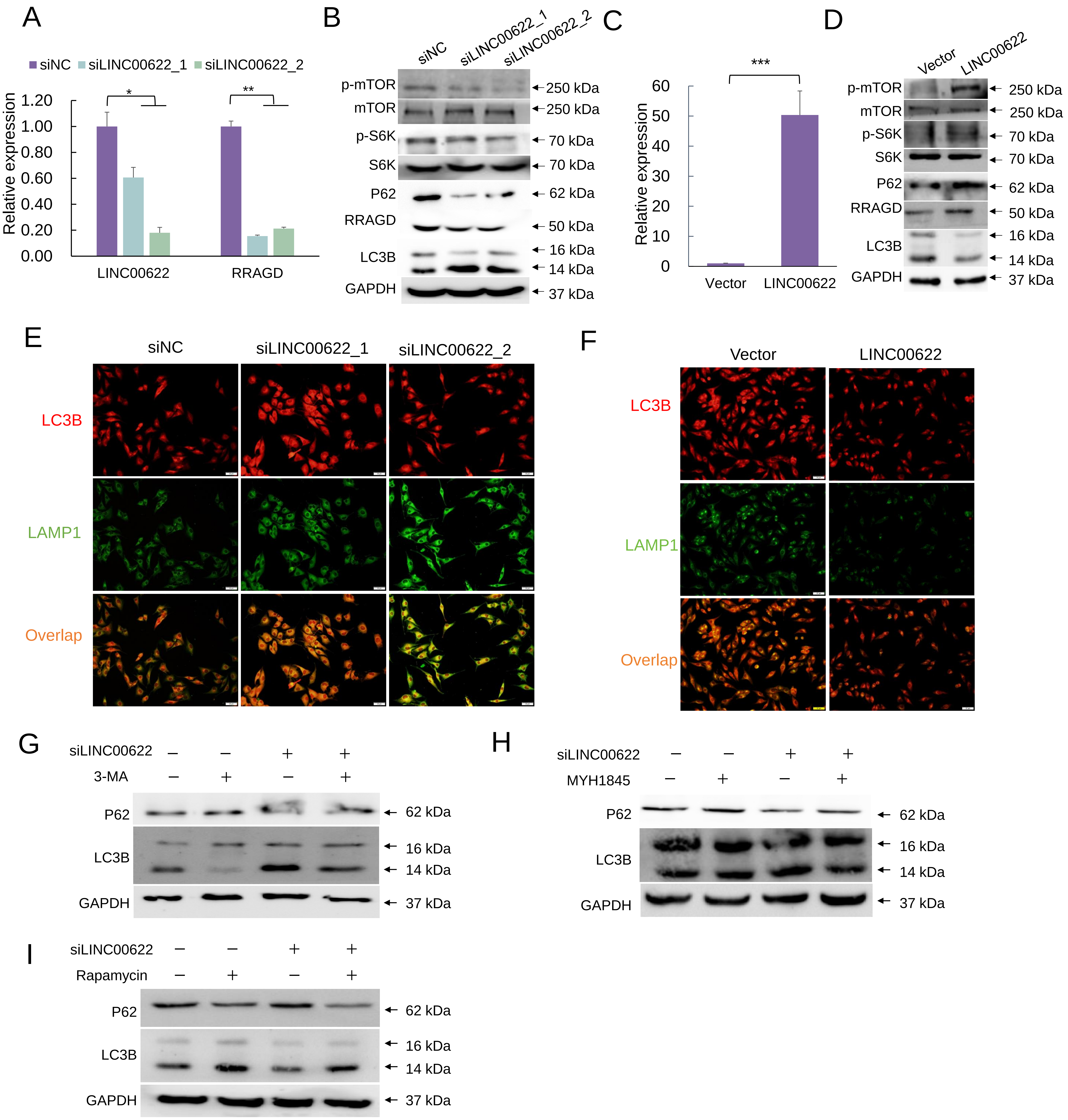
A
siNC
siLINC00622_1
siLINC00622_2
Relative expression
1.20
1.00
0.80
0.60
0.40
0.20
0.00
*
**
LINC00622
RRAGD
B
siNC
siLINC00622_1
siLINC00622_2
p-mTOR
mTOR
p-S6K
S6K
P62
RRAGD
LC3B
GAPDH
250 kDa
250 kDa
70 kDa
70 kDa
62 kDa
50 kDa
16 kDa
14 kDa
37 kDa
C
***
Relative expression
60
50
40
30
20
10
0
Vector
LINC00622
D
Vector
LINC00622
p-mTOR
mTOR
p-S6K
S6K
P62
RRAGD
LC3B
GAPDH
250 kDa
250 kDa
70 kDa
70 kDa
62 kDa
50 kDa
16 kDa
14 kDa
37 kDa
E
siNC
siLINC00622_1
siLINC00622_2
LC3B
LAMP1
Overlap
F
Vector
LINC00622
LC3B
LAMP1
Overlap
G
siLINC00622 – – + +
3-MA – + – +
P62
LC3B
GAPDH
62 kDa
16 kDa
14 kDa
37 kDa
H
siLINC00622 – – + +
MYH1845 – + – +
P62
LC3B
GAPDH
62 kDa
16 kDa
14 kDa
37 kDa
I
siLINC00622 – – + +
Rapamycin – + – +
P62
LC3B
GAPDH
62 kDa
16 kDa
14 kDa
37 kDa

Figure S3. LINC00622 regulates mTORC1-modulated autophagy through RRAGD. (**A**) LINC00622 and RRAGD RNA was detected by qPCR after knockdown of LINC00622 in A375 cells. (**B**) Western blot detection of the levels of RRAGD, p-mTOR, p-S6K, LC3B-II and P62 after LINC00622 depletion in A375 cells. (**C**) qPCR detection of LINC00622 expression after LINC00622 overexpression in A375 cells. (**D**) Western blot detection of the levels of RRAGD, p-mTOR, p-S6K, LC3B-II and P62 after LINC00622 overexpression in A375 cells. (**E**) IF detection of LC3B foci after LINC00622 depletion in melanoma cells. (**F**) IF detection of LC3B foci after LINC00622 overexpression in melanoma cells. The role of LINC00622 in regulating autophagy was validated using autophagy inhibitors 3-MA and MYH1485 treatments in melanoma cells following LINC00622 silencing by Western blot detection of LC3B and P62 in A375 cells (**G, H**). The role of LINC00622 in regulating autophagy was validated using autophagy activator Rapamycin following LINC00622 overexpression by Western blot detection of LC3B and P62 in A375 cells (**I**). Scale bar, 50 μM.

Figure S4

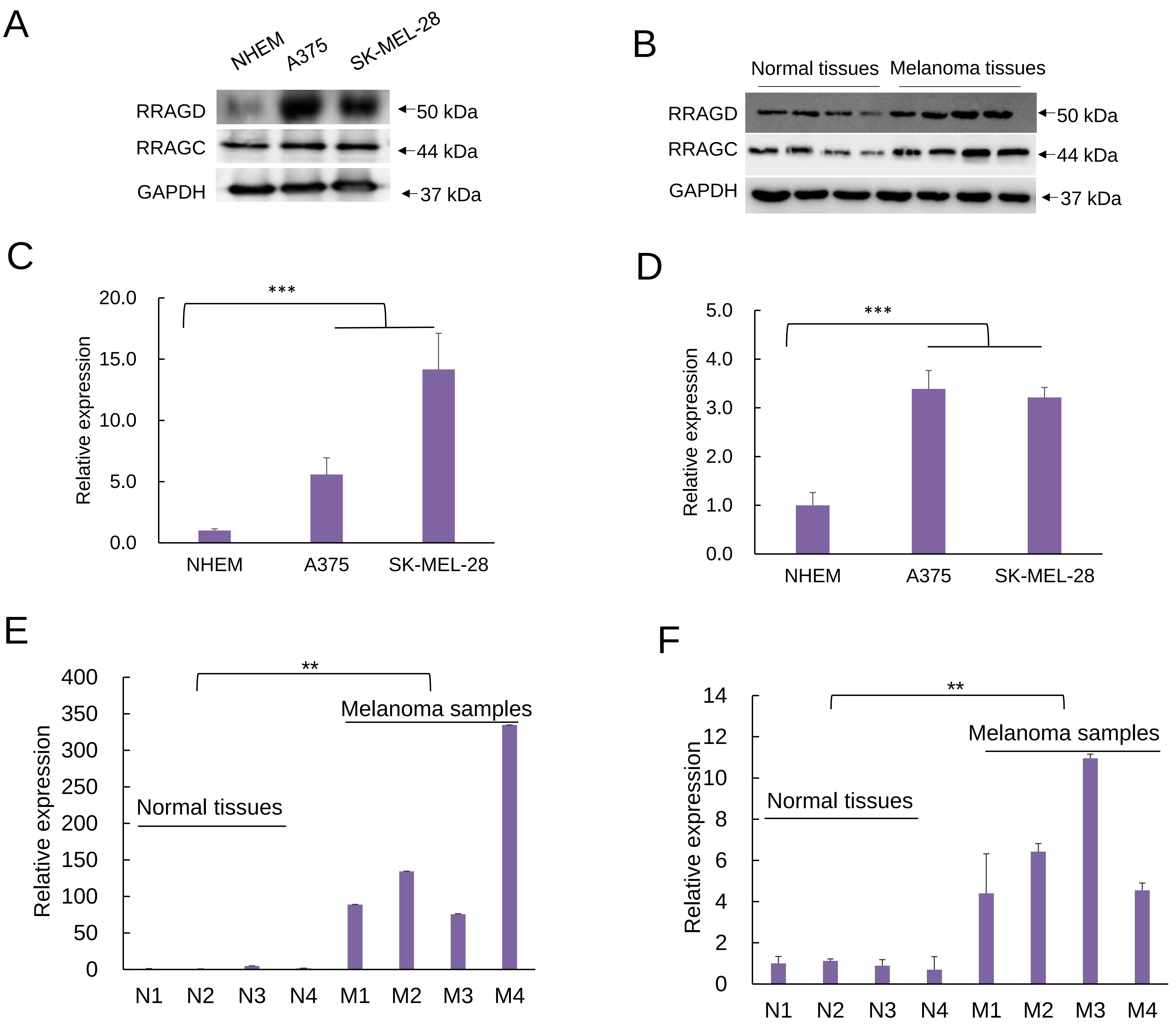

Figure S4. Detection of the protein and mRNA expression of RRAGD and RRAGC in melanoma. (**A**) The expression levels of RRAGD and RRAGC were detected by Western blot in melanoma cell lines (A375 and SK-MEL-28) compared with the primary normal human epidermal keratinocytes (NHEK). (**B**) The expression levels of RRAGD and RRAGC were detected by Western blot between normal cutaneous tissues and melanoma tumors. The expression levels of RRAGD (**C**) and RRAGC (**D**) were detected by qPCR in melanoma cell lines (A375 and SK-MEL-28) compared with the primary normal human epidermal keratinocytes (NHEK). The expression levels of RRAGD (**E**) and RRAGC (**F**) were detected by qPCR between normal cutaneous tissues and melanoma tumors.

Figure S5

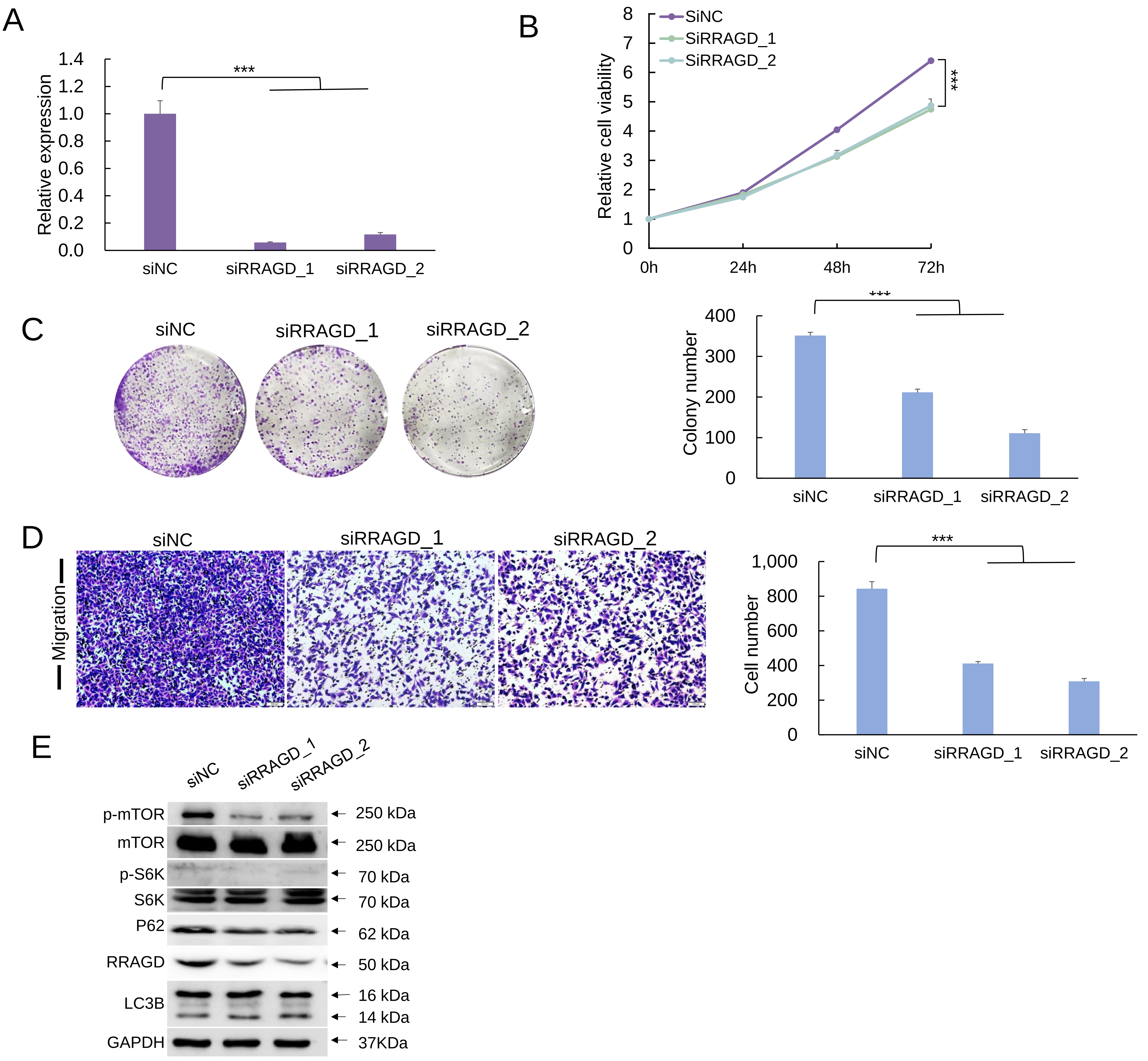

Figure S5. LINC00622 regulates mTORC1-regulated autophagy through RRAGD. (**A**) RRAGD RNA expression was detected by qPCR after knockdown of RRAGD by RNA interference in A375 cells. Measurement of cell proliferation by CCK-8 assay (**B**), colony formation assay (**C**), Transwell migration assay (**D**) were performed in A375 cells after RRAGD silencing. (**E**) RRAGD, p-mTOR, p-S6K, LC3B and P62 were detected by Western blot in melanoma cells after RRAGD depletion in A375 cells.

Figure S6

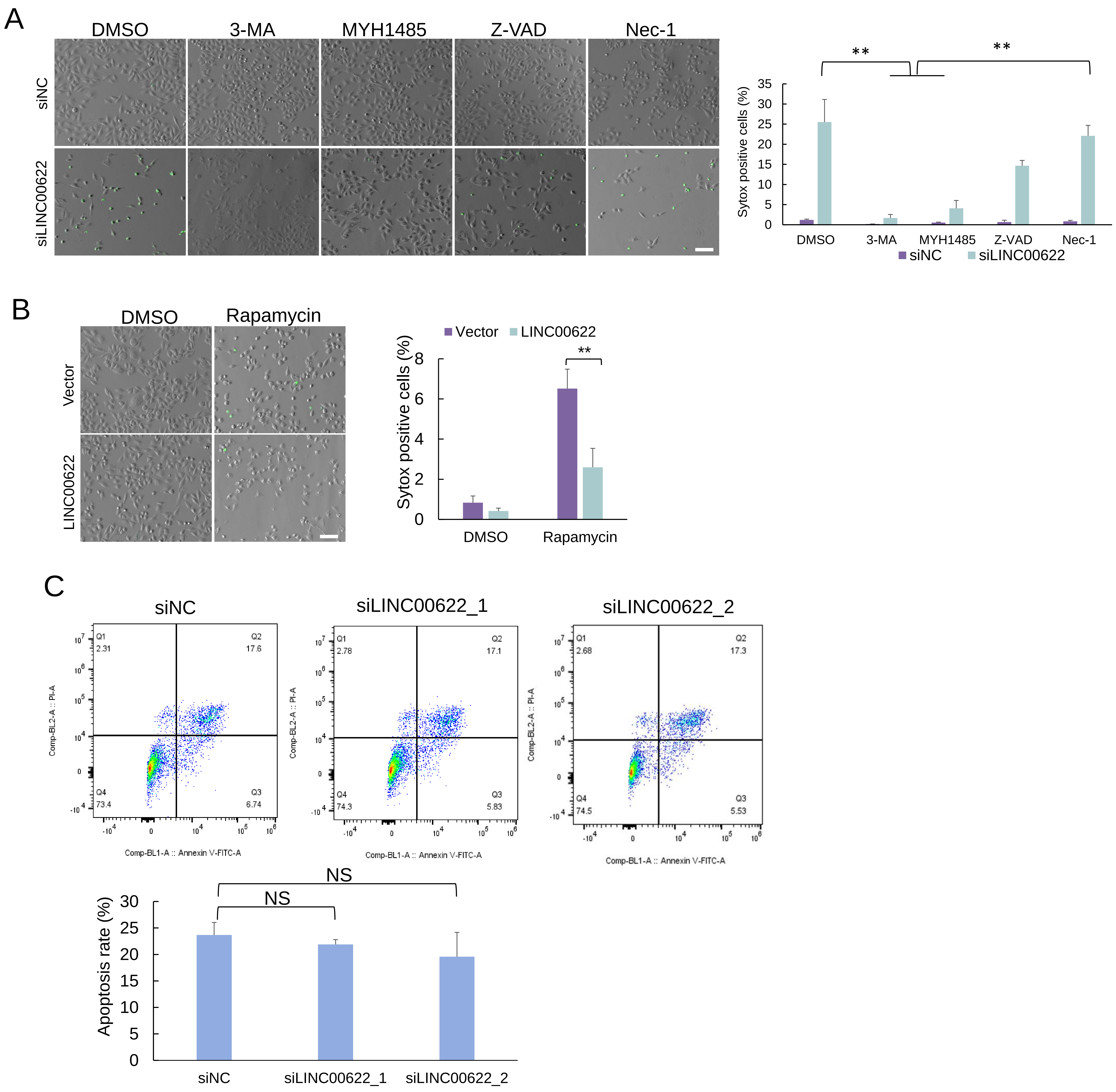

Figure S6. LINC00622 represses autophagic cell death. (**A**) Sytox green staining was performed to detect the major type of cell death induced by LINC00622 depletion in A375 melanoma cells with 3-MA, MYH1485, Z-VAD-FMK or Nec-1 treatments. Scale bar, 100 μM. (**B**) Evaluate the role of LINC00622 in repressing Rapamycin-induced cell death in A375 melanoma cells by Sytox green staining. Scale bar, 100 μM. (**C**) fluorescence-activated cell sorting (FACS) analysis detected apoptosis rate in SK-MEL-28 melanoma cells after LINC00622 depletion.

Figure S7

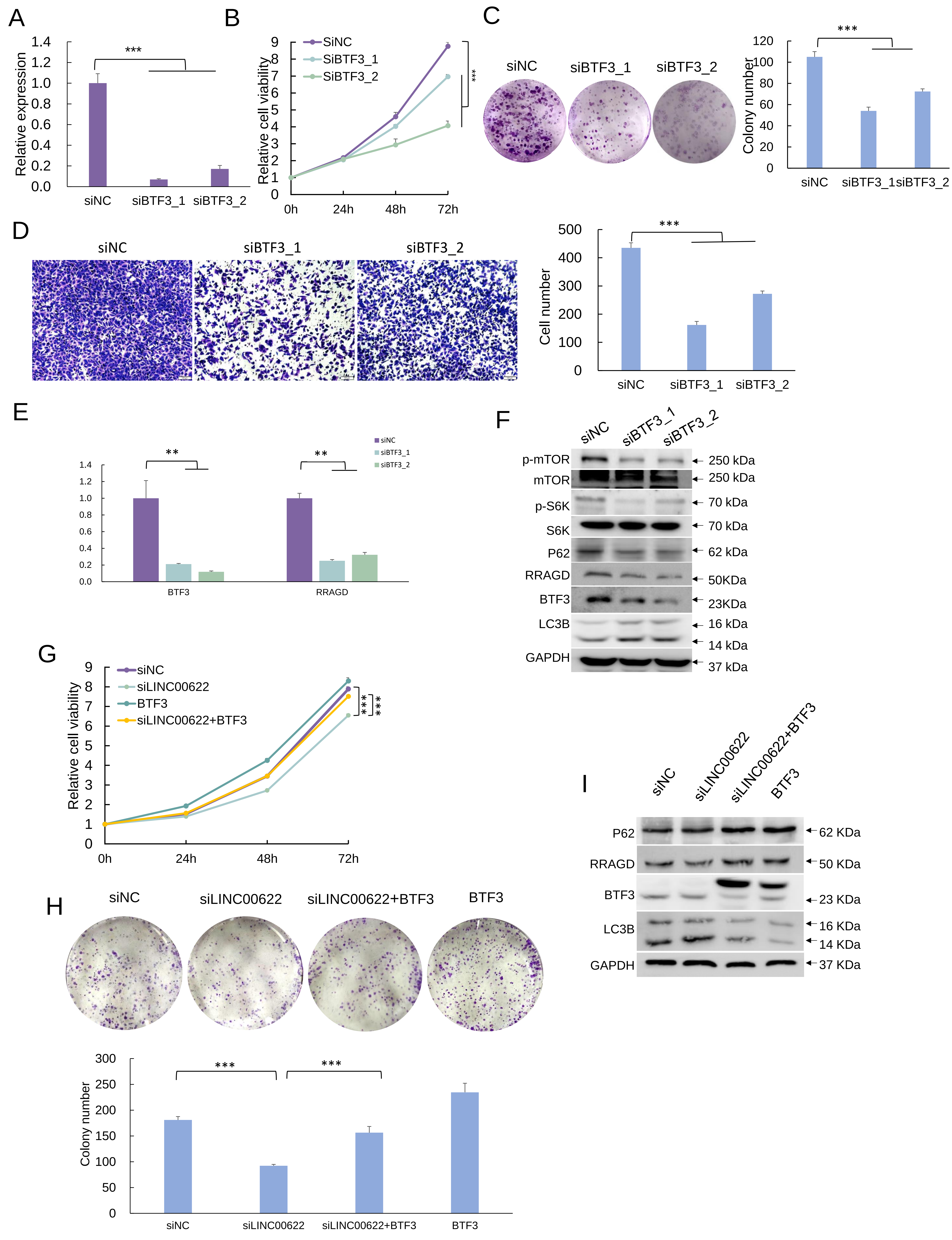
A
Relative expression
1.4
1.2
1.0
0.8
0.6
0.4
0.2
0.0
***
siNC
siBTF3_1
siBTF3_2
B
Relative cell viability
SiNC
SiBTF3_1
SiBTF3_2
***
0h
24h
48h
72h
C
siNC
siBTF3_1
siBTF3_2
Colony number
***
120
100
80
60
40
20
0
siNC
siBTF3_1siBTF3_2
D
siNC
siBTF3_1
siBTF3_2
Cell number
***
500
400
300
200
100
0
siNC
siBTF3_1
siBTF3_2
E
**
**
siNC
siBTF3_1
siBTF3_2
BTF3
RRAGD
F
siNC
siBTF3_1
siBTF3_2
p-mTOR
250 kDa
mTOR
250 kDa
p-S6K
70 kDa
S6K
70 kDa
P62
62 kDa
RRAGD
50KDa
BTF3
23KDa
LC3B
16 kDa
14 kDa
GAPDH
37 kDa
G
Relative cell viability
siNC
siLINC00622
BTF3
siLINC00622+BTF3
***
***
0h
24h
48h
72h
H
siNC
siLINC00622
siLINC00622+BTF3
BTF3
Colony number
***
***
300
250
200
150
100
50
0
siNC
siLINC00622
siLINC00622+BTF3
BTF3
I
siNC
siLINC00622
siLINC00622+BTF3
BTF3
P62
62 KDa
RRAGD
50 KDa
BTF3
23 KDa
LC3B
16 KDa
14 KDa
GAPDH
37 KDa

Figure S7. The repression of RRAGD-modulated autophagy is dependent on BTF3. (**A**) BTF3 RNA expression was detected by qPCR after knockdown of BTF3 by RNA interference in A375 melanoma cells. Measurement of cell proliferation by CCK-8 assay (**B**), colony formation assay (**C**), Transwell migration assay (**D**) were performed in A375 melanoma cells treated with RNA interference to deplete BTF3. (**E**) BTF3 and RRAGD were detected by qPCR after knockdown of BTF3 in A375 melanoma cells. (**F**) BTF3, RRAGD, p-mTOR, p-S6K, LC3B and P62 were detected by Western blot in melanoma cells after LINC00622 depletion in A375 melanoma cells. (**H**) The decreased proliferative capacity and (**I**) colony formation in response to silencing of LINC00622 could be rescued by overexpression of BTF3 in A375 melanoma cells. (**J**)The enhanced LC3-II generation together with decreased P62 and RRAGD levels after LINC00622 depletion could be significantly tuned back by BTF3 overexpression in A375 melanoma cells.
