# Supplementary material for: LINC00622 transcriptionally promotes RRAGD to repress mTORC1-modulated autophagic cell death by associating with BTF3 in cutaneous melanoma
Source: Cell Death Dis. 2025 Jul 12;16(1):515. doi: 10.1038/s41419-025-07828-1 (PMC12255717; doi:10.1038/s41419-025-07828-1)
Supplement: Supplementary file 2 — Supplementary Original Data [file 41419_2025_7828_MOESM2_ESM.pdf]

Fig.4B

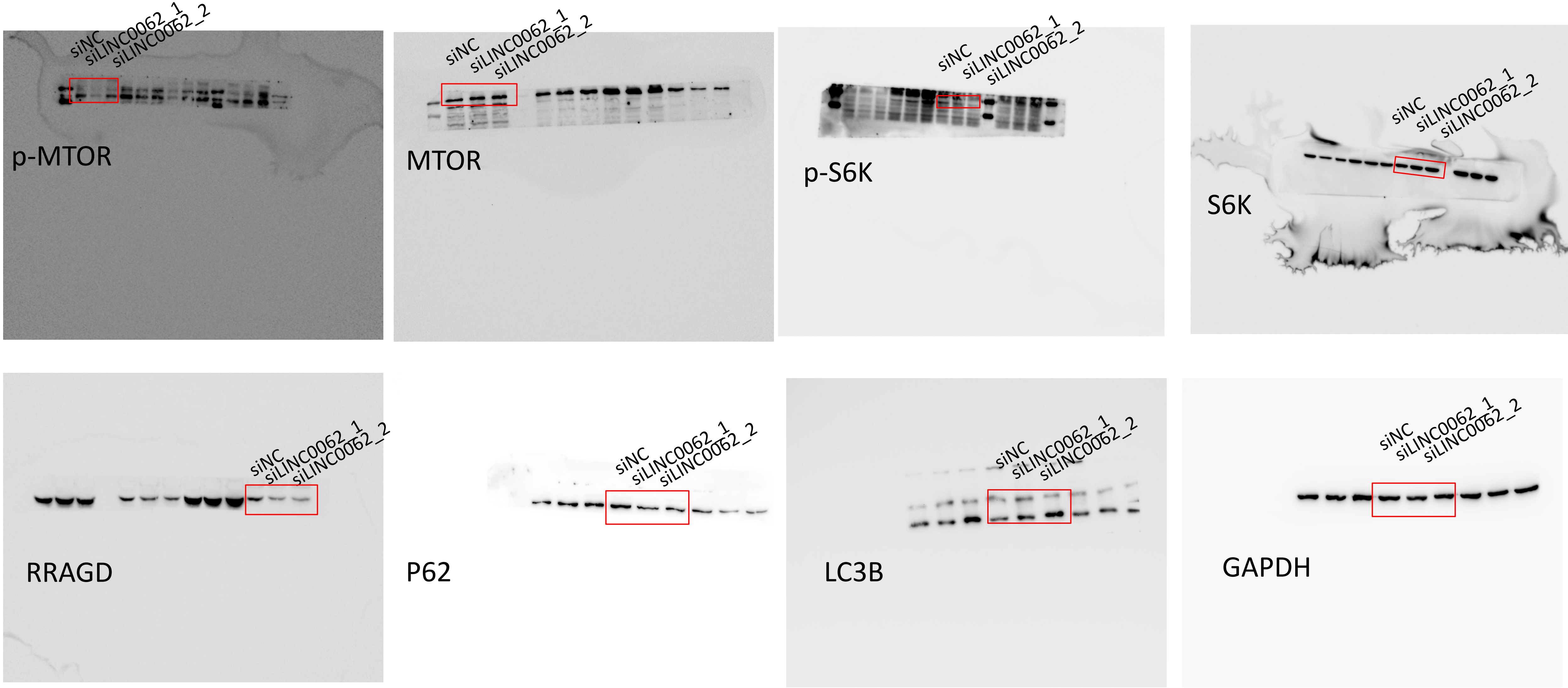
siNC
siLINC0062_1
siLINC0062_2
p-MTOR
siNC
siLINC0062_1
siLINC0062_2
MTOR
siNC
siLINC0062_1
siLINC0062_2
p-S6K
siNC
siLINC0062_1
siLINC0062_2
S6K
siNC
siLINC0062_1
siLINC0062_2
RRAGD
siNC
siLINC0062_1
siLINC0062_2
P62
siNC
siLINC0062_1
siLINC0062_2
LC3B
siNC
siLINC0062_1
siLINC0062_2
GAPDH

Fig.4D

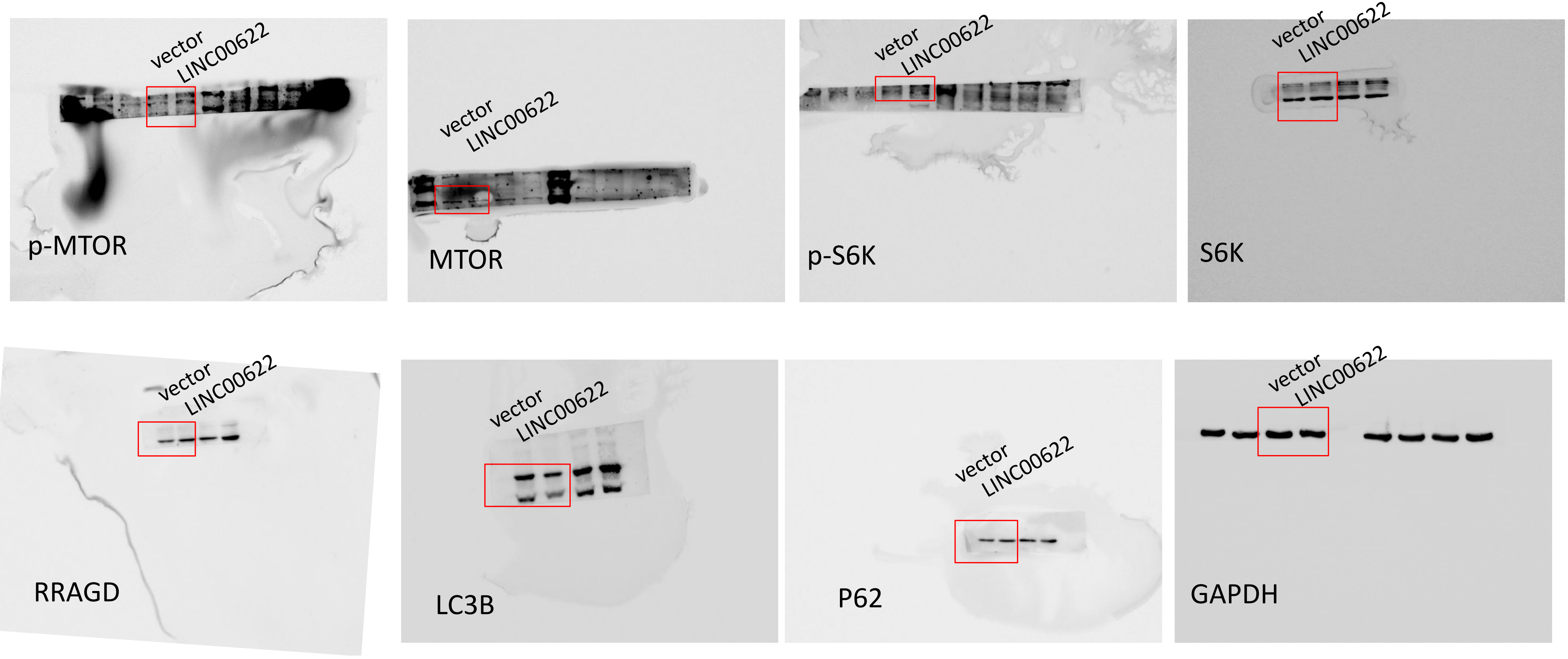

vector
LINC00622
p-MTOR
vector
LINC00622
MTOR
vetor
LINC00622
p-S6K
vector
LINC00622
S6K
vector
LINC00622
RRAGD
vector
LINC00622
LC3B
vector
LINC00622
P62
vector
LINC00622
GAPDH

Fig.4E

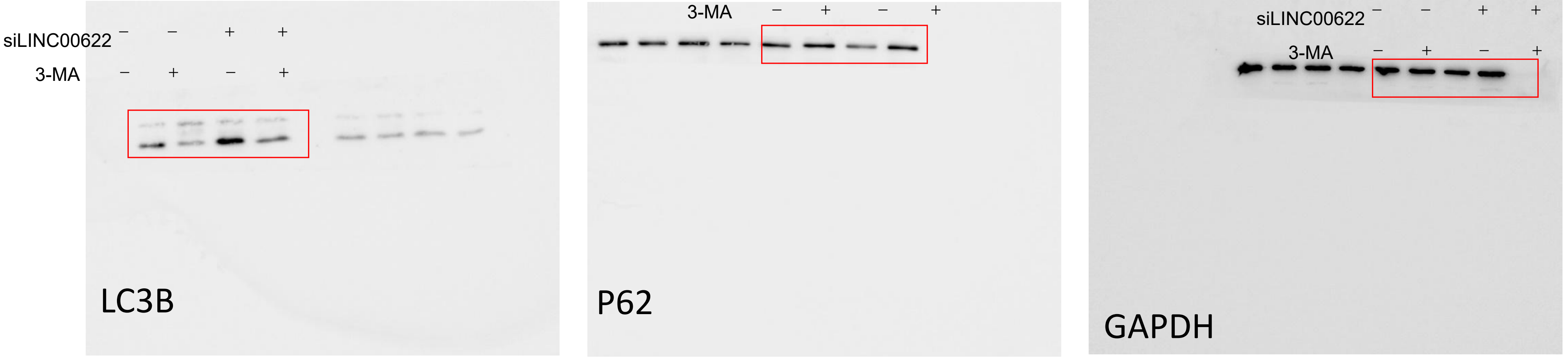
siLINC00622 – – + +
3-MA – + – +
LC3B
siLINC00622 – – + +
3-MA – + – +
P62
siLINC00622 – – + +
3-MA – + – +
GAPDH

Fig.4F

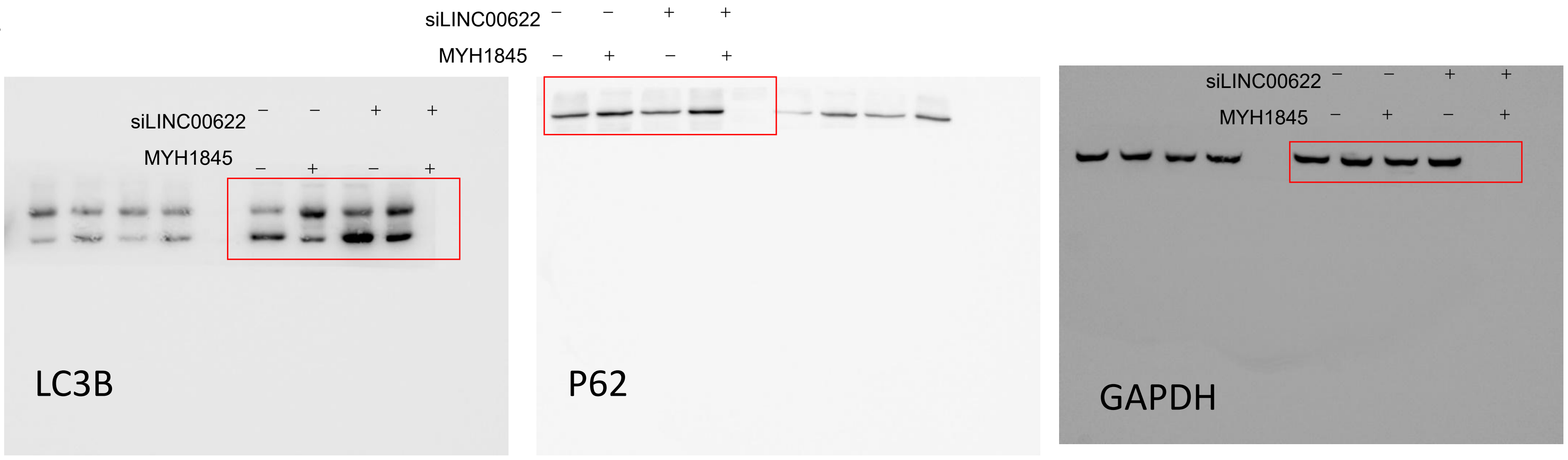
siLINC00622 – – + +
MYH1845 – + – +
LC3B
siLINC00622 – – + +
MYH1845 – + – +
P62
siLINC00622 – – + +
MYH1845 – + – +
GAPDH

Fig.4H

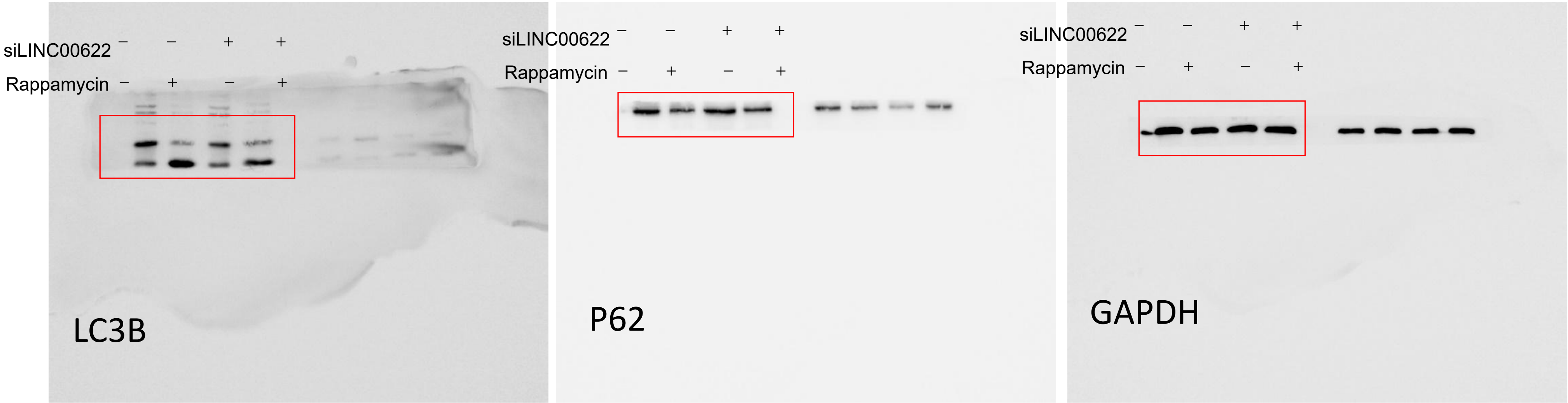
siLINC00622 − − + +
Rappamycin − + − +
LC3B
siLINC00622 − − + +
Rappamycin − + − +
P62
siLINC00622 − − + +
Rappamycin − + − +
GAPDH

Fig.5E

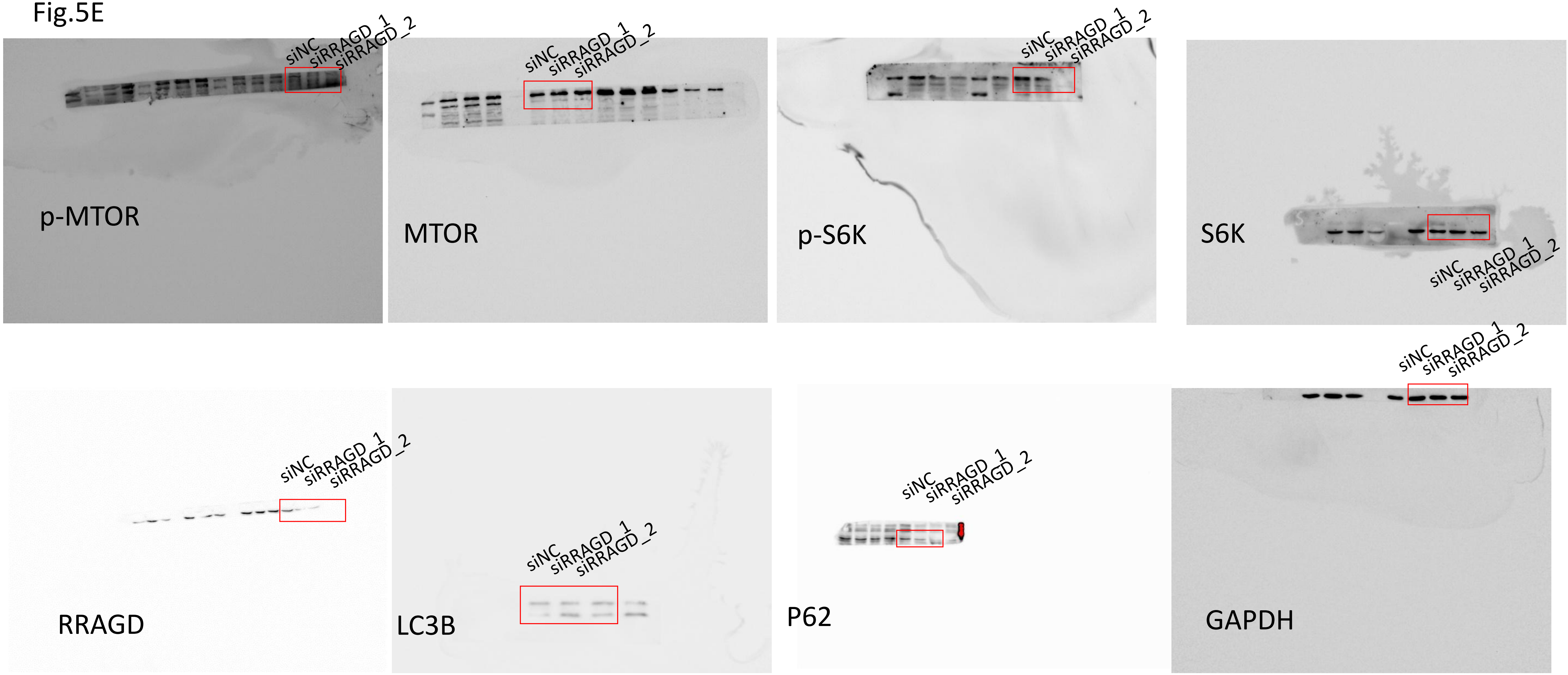
siNC
siRRAGD_1
siRRAGD_2
p-MTOR
MTOR
p-S6K
S6K
RRAGD
LC3B
P62
GAPDH

Fig.6B

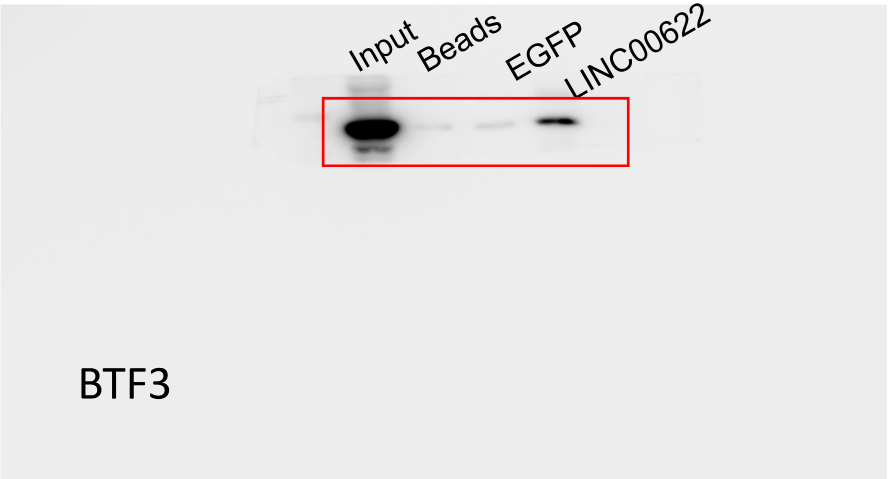
Input
Beads
EGFP
LINC00622
BTF3

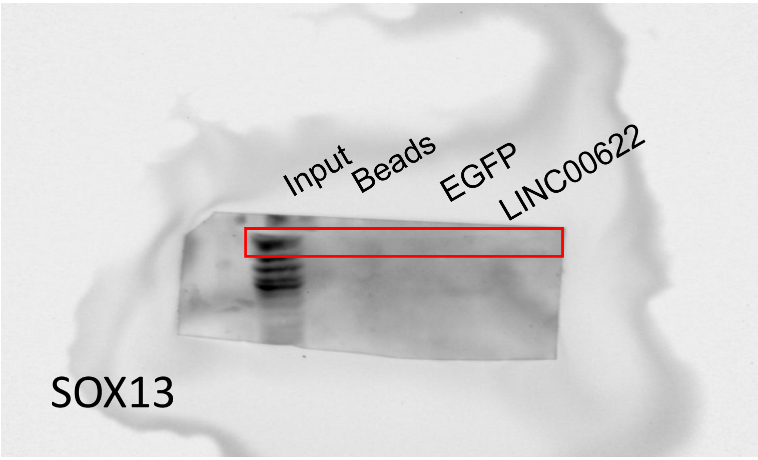
Input
Beads
EGFP
LINC00622
SOX13

Fig.6F

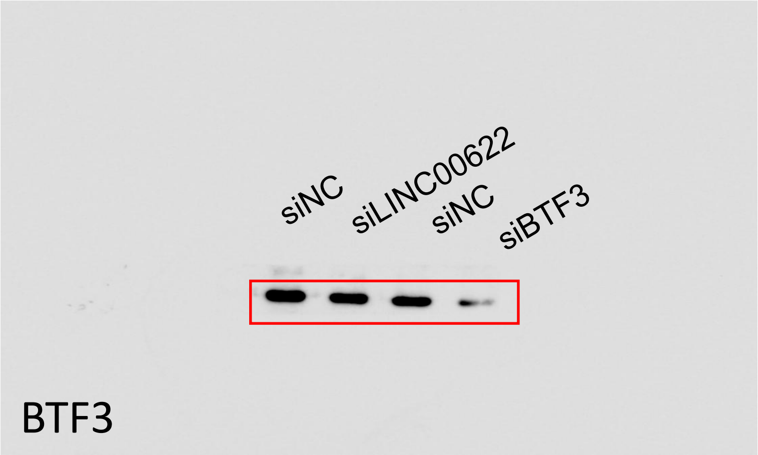
siNC
siLINC00622
siNC
siBTF3
BTF3

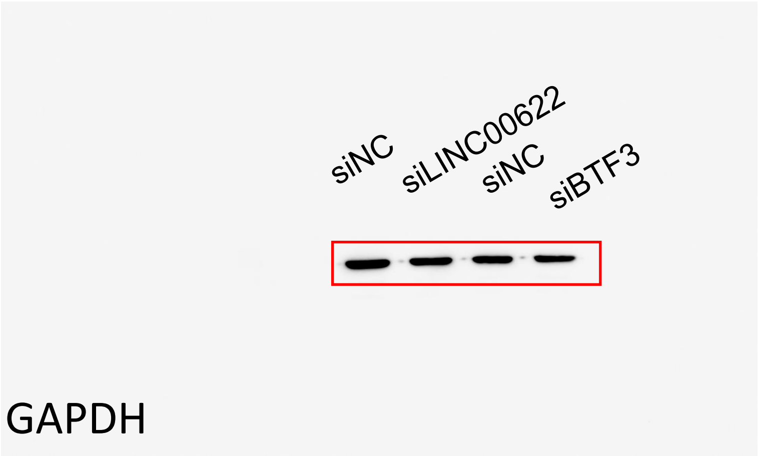
siNC
siLINC00622
siNC
siBTF3
GAPDH

Fig.7F

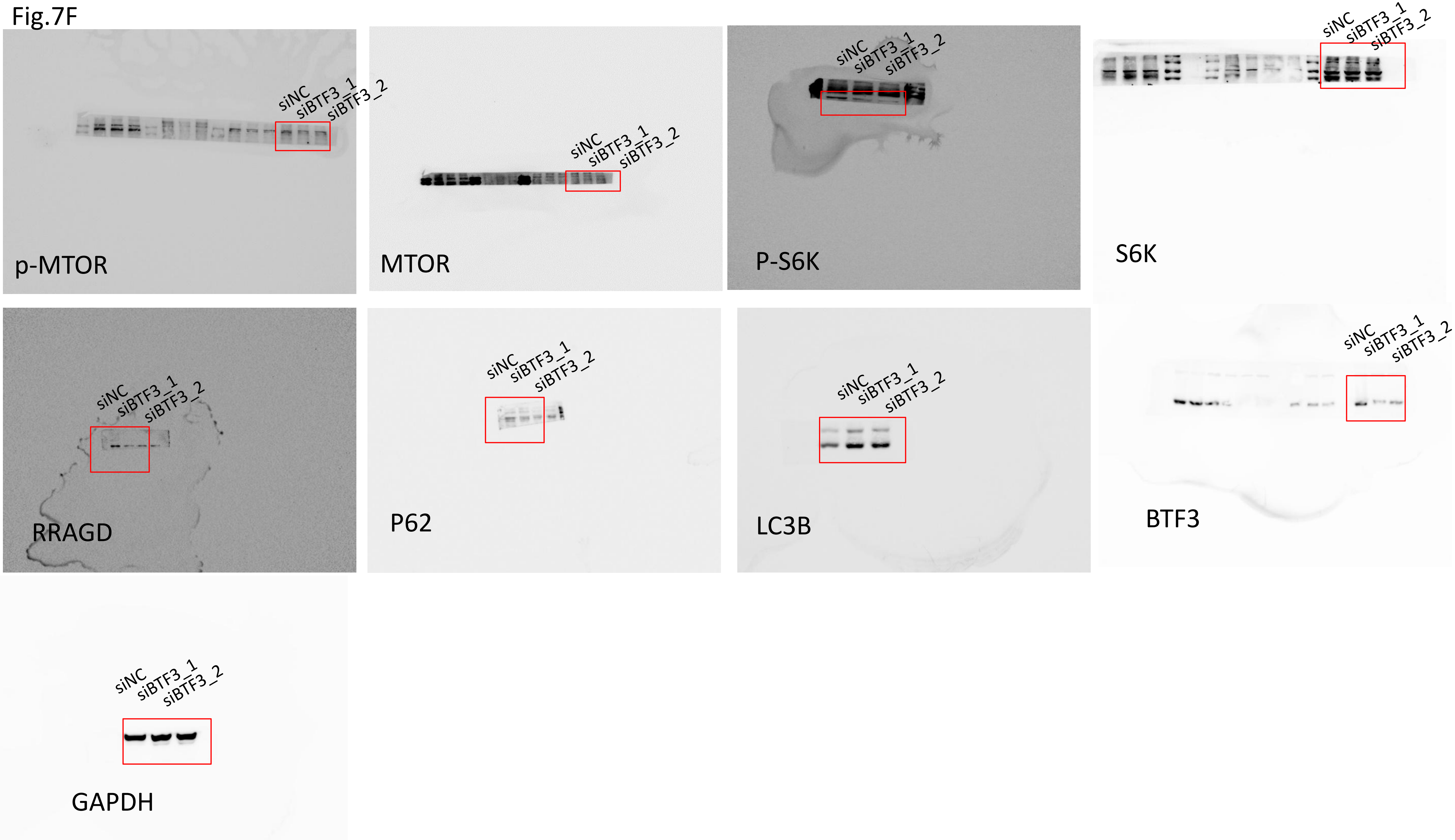
siNC
siBTF3_1
siBTF3_2
p-MTOR
siNC
siBTF3_1
siBTF3_2
MTOR
siNC
siBTF3_1
siBTF3_2
P-S6K
siNC
siBTF3_1
siBTF3_2
S6K
siNC
siBTF3_1
siBTF3_2
RRAGD
siNC
siBTF3_1
siBTF3_2
P62
siNC
siBTF3_1
siBTF3_2
LC3B
siNC
siBTF3_1
siBTF3_2
BTF3
siNC
siBTF3_1
siBTF3_2
GAPDH

Fig. 7I

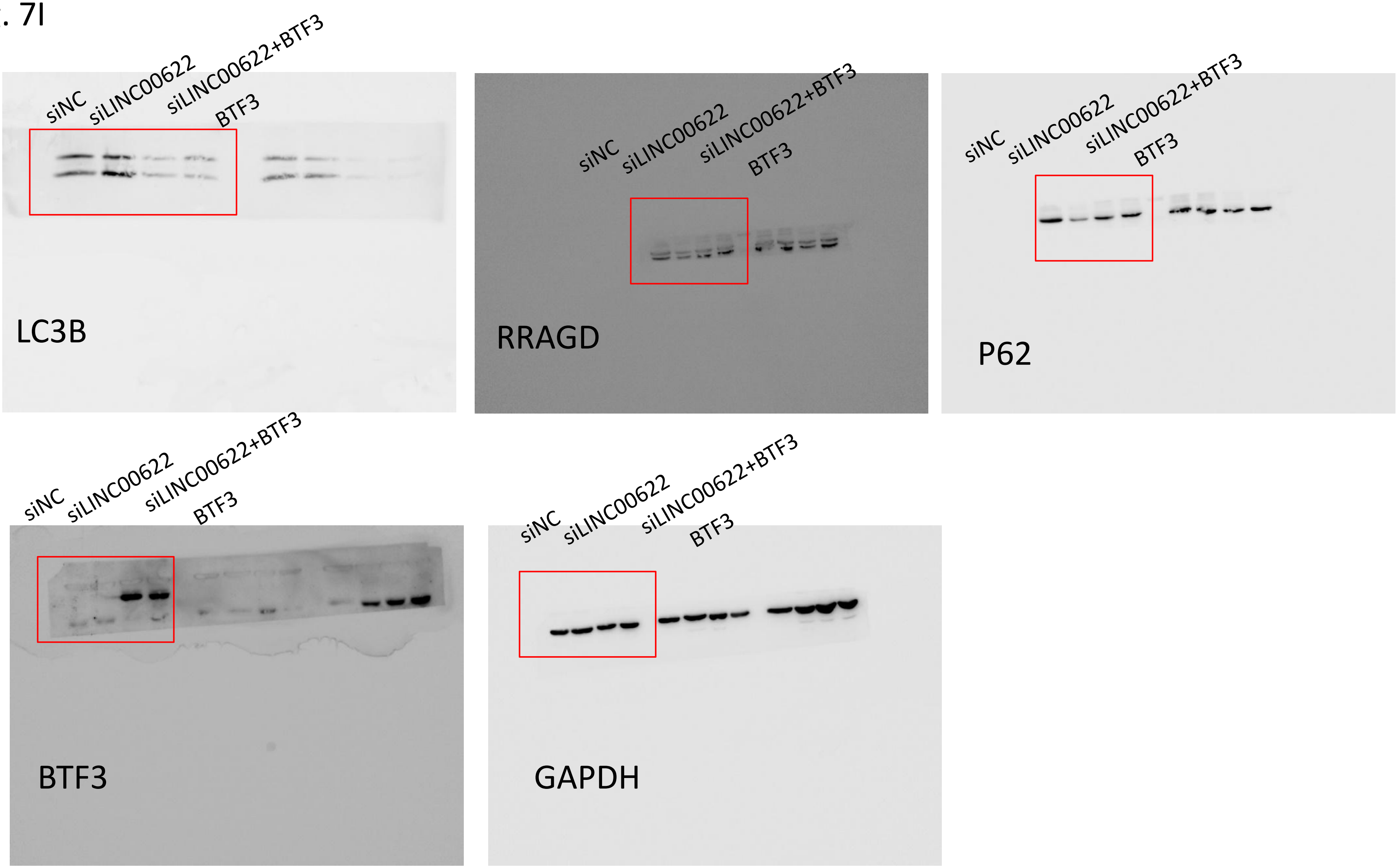
siNC
siLINC00622
siLINC00622+BTF3
BTF3
LC3B
siNC
siLINC00622
siLINC00622+BTF3
BTF3
RRAGD
siNC
siLINC00622
siLINC00622+BTF3
BTF3
P62
siNC
siLINC00622
siLINC00622+BTF3
BTF3
BTF3
siNC
siLINC00622
siLINC00622+BTF3
BTF3
GAPDH

Fig. 8G

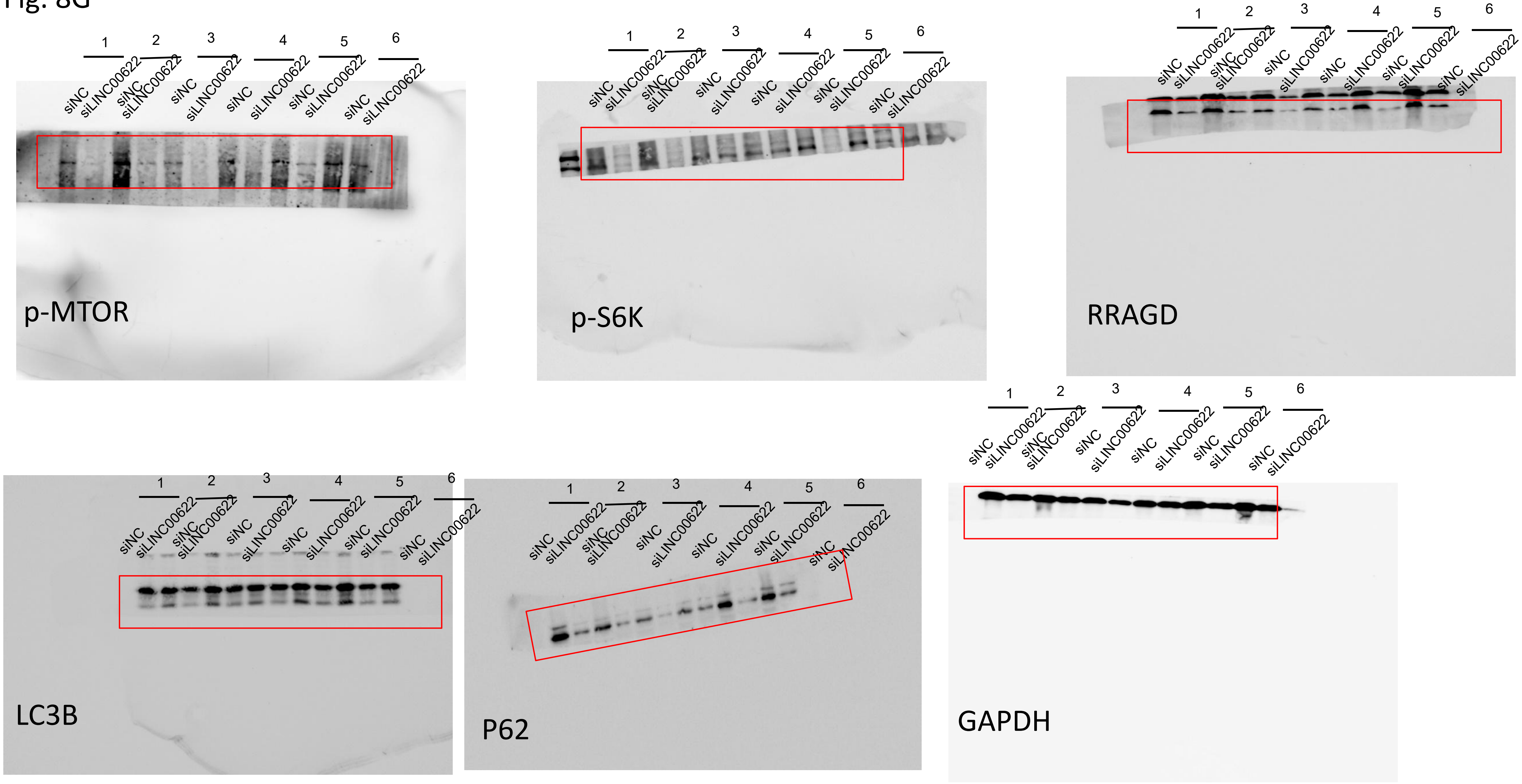

Fig.S3B

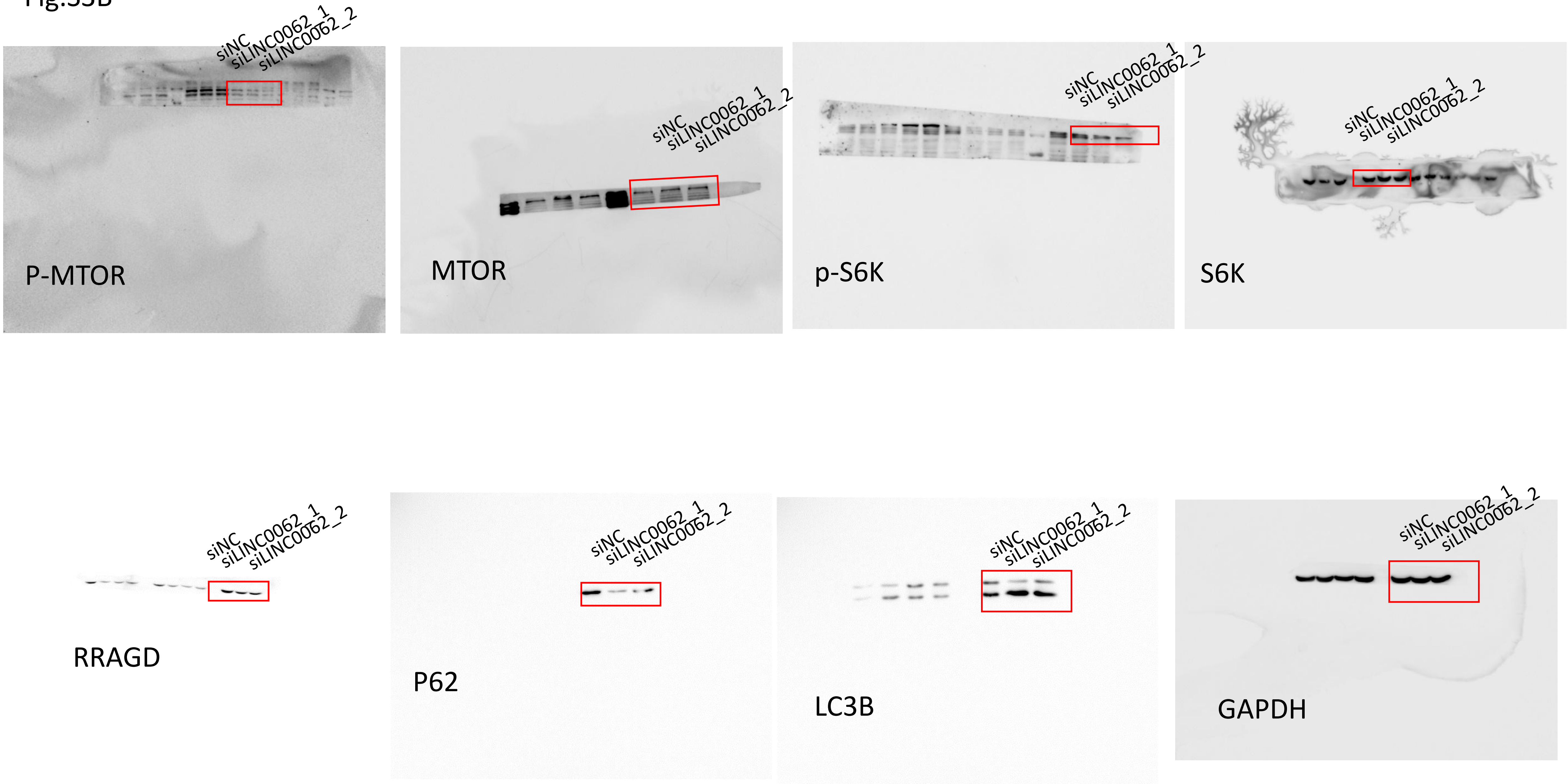
siNC
siLINC0062_1
siLINC0062_2
P-MTOR
MTOR
p-S6K
S6K
RRAGD
P62
LC3B
GAPDH

Fig.S3D

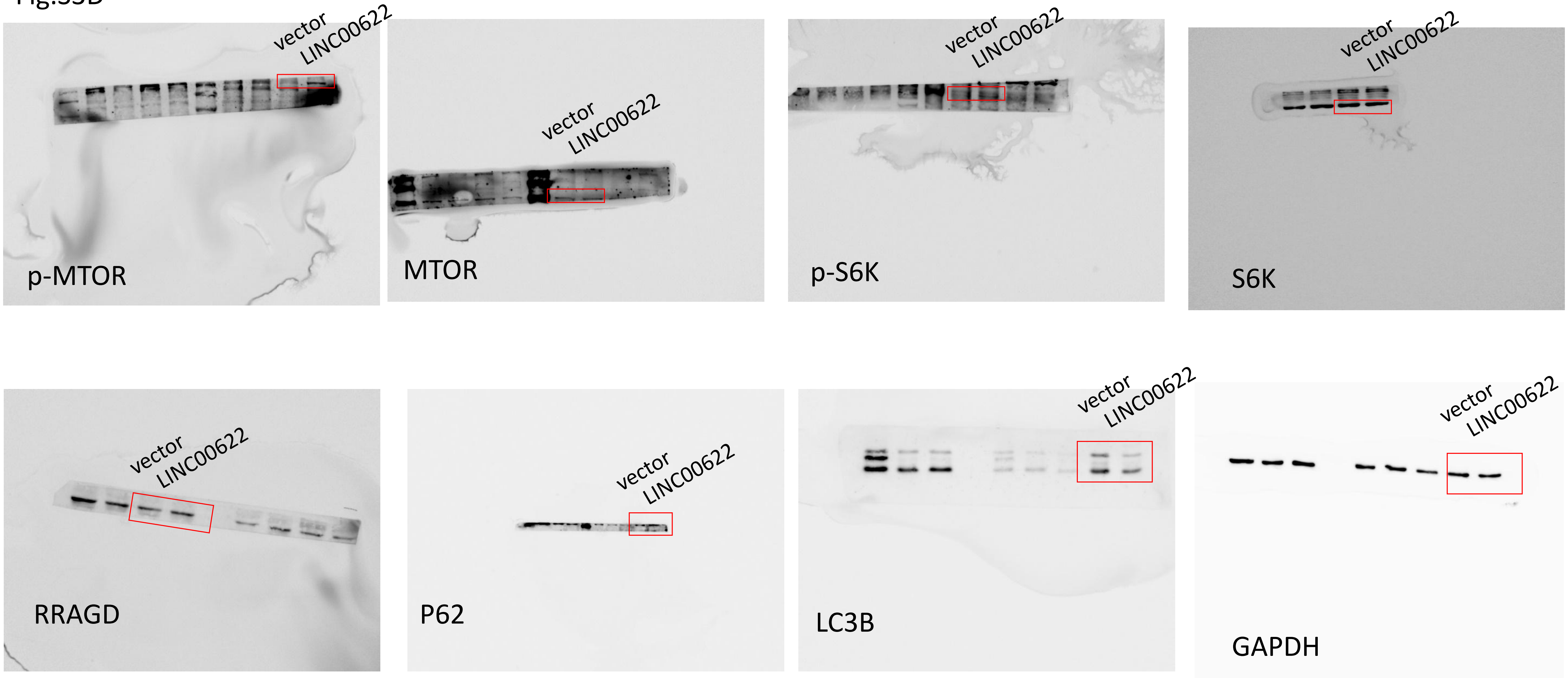

vector
LINC00622
p-MTOR
vector
LINC00622
MTOR
vector
LINC00622
p-S6K
vector
LINC00622
S6K
vector
LINC00622
RRAGD
vector
LINC00622
P62
vector
LINC00622
LC3B
vector
LINC00622
GAPDH

Fig.S3G

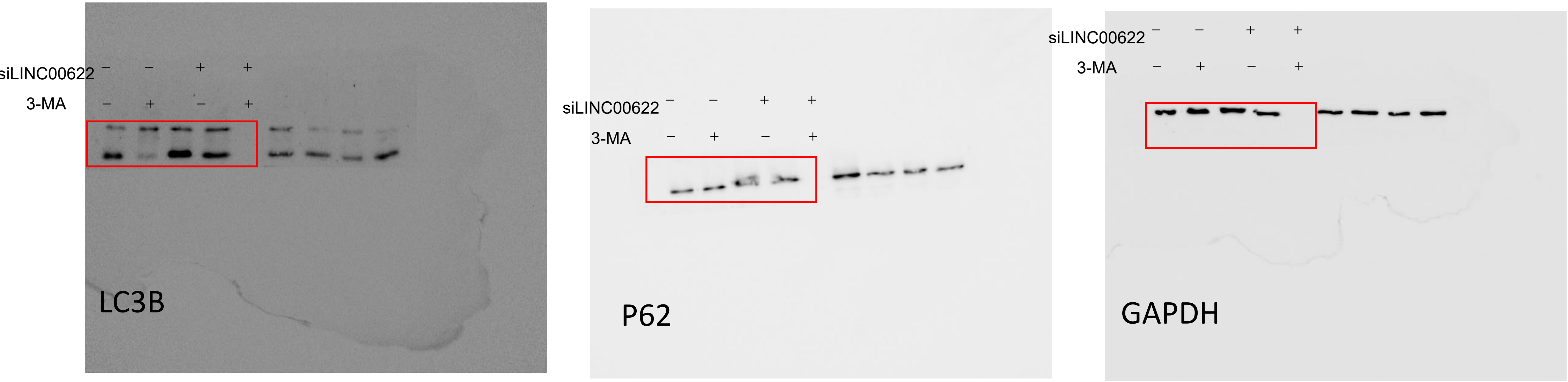

Fig.S3H

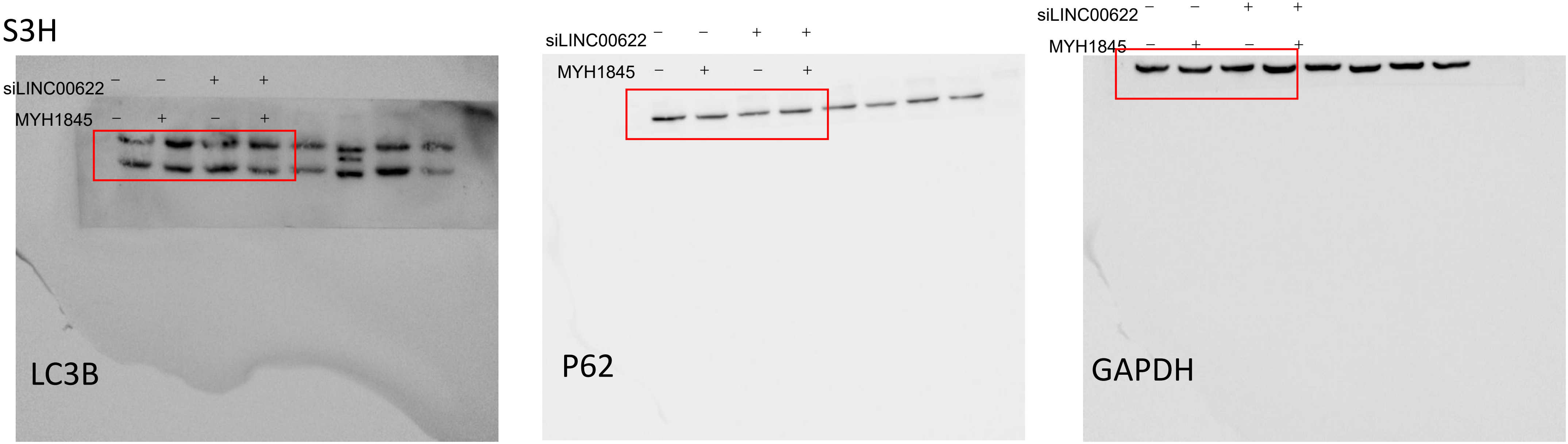

Fig.S3I

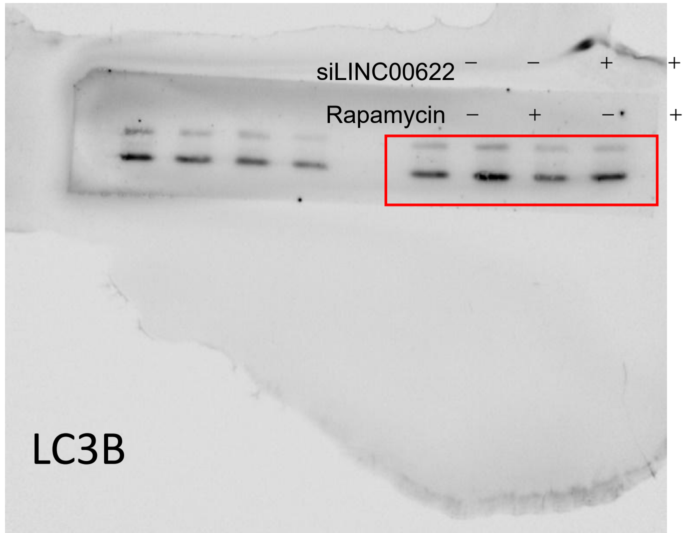
siLINC00622 − − + +
Rapamycin − + − +
LC3B

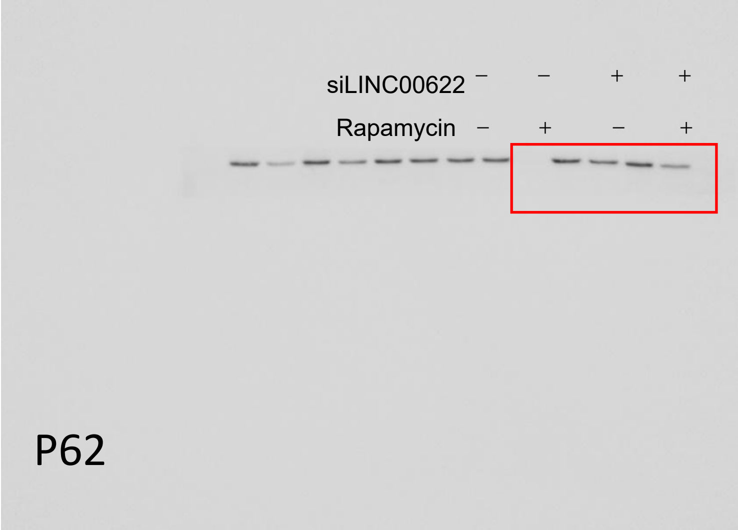
siLINC00622 − − + +
Rapamycin − + − +
P62

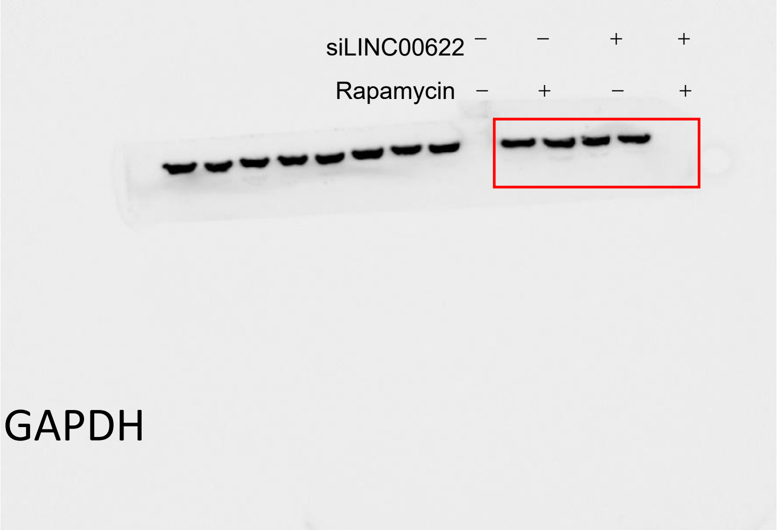
siLINC00622 − − + +
Rapamycin − + − +
GAPDH

Fig.S4A

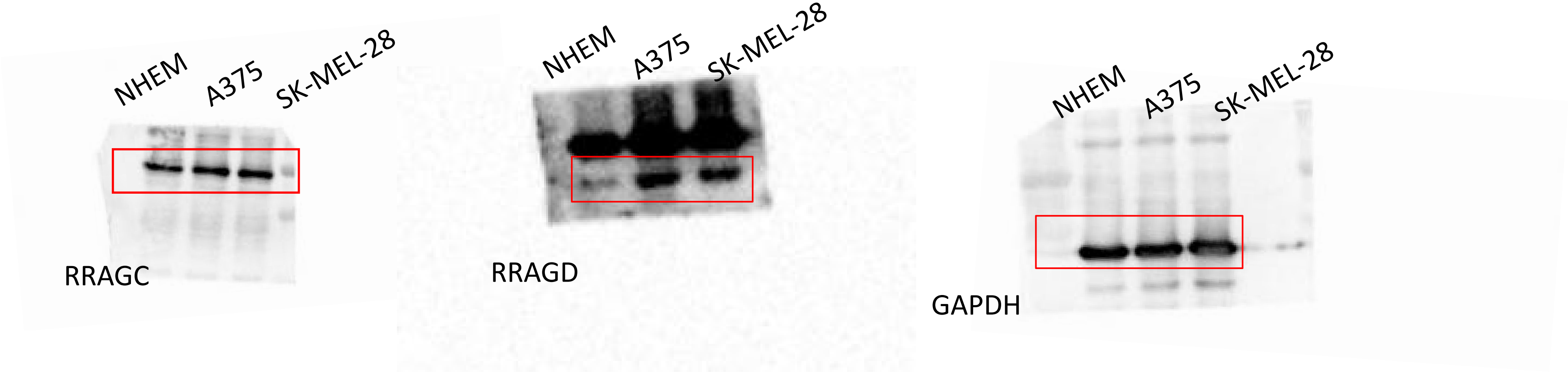

Fig.S4B

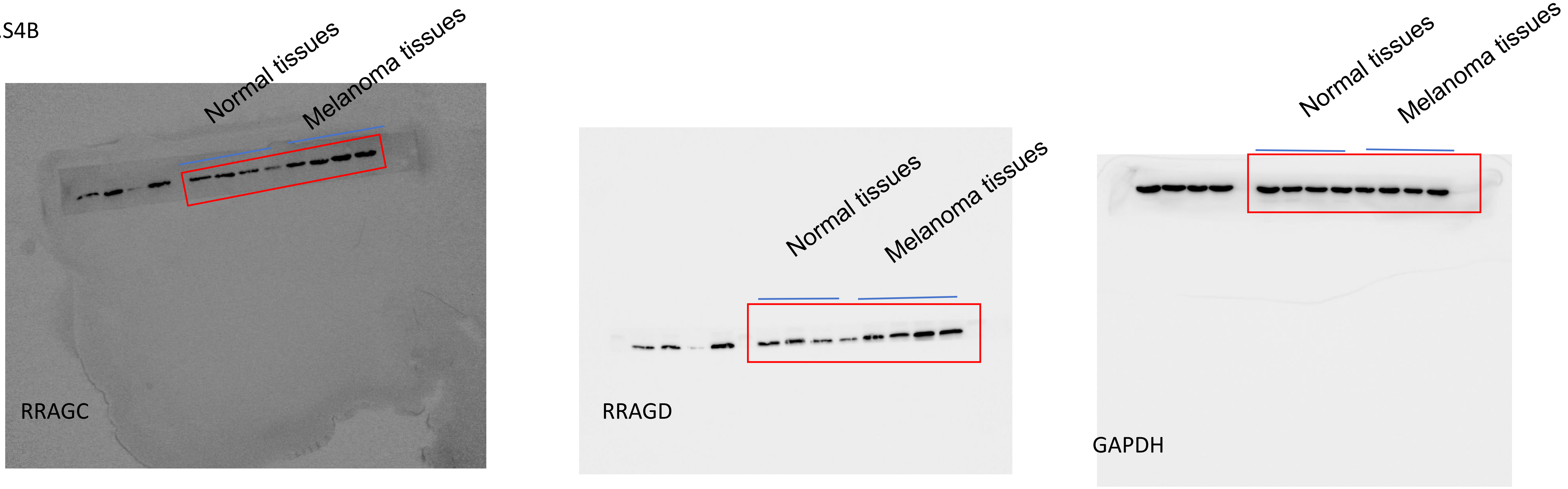

Fig.S5E

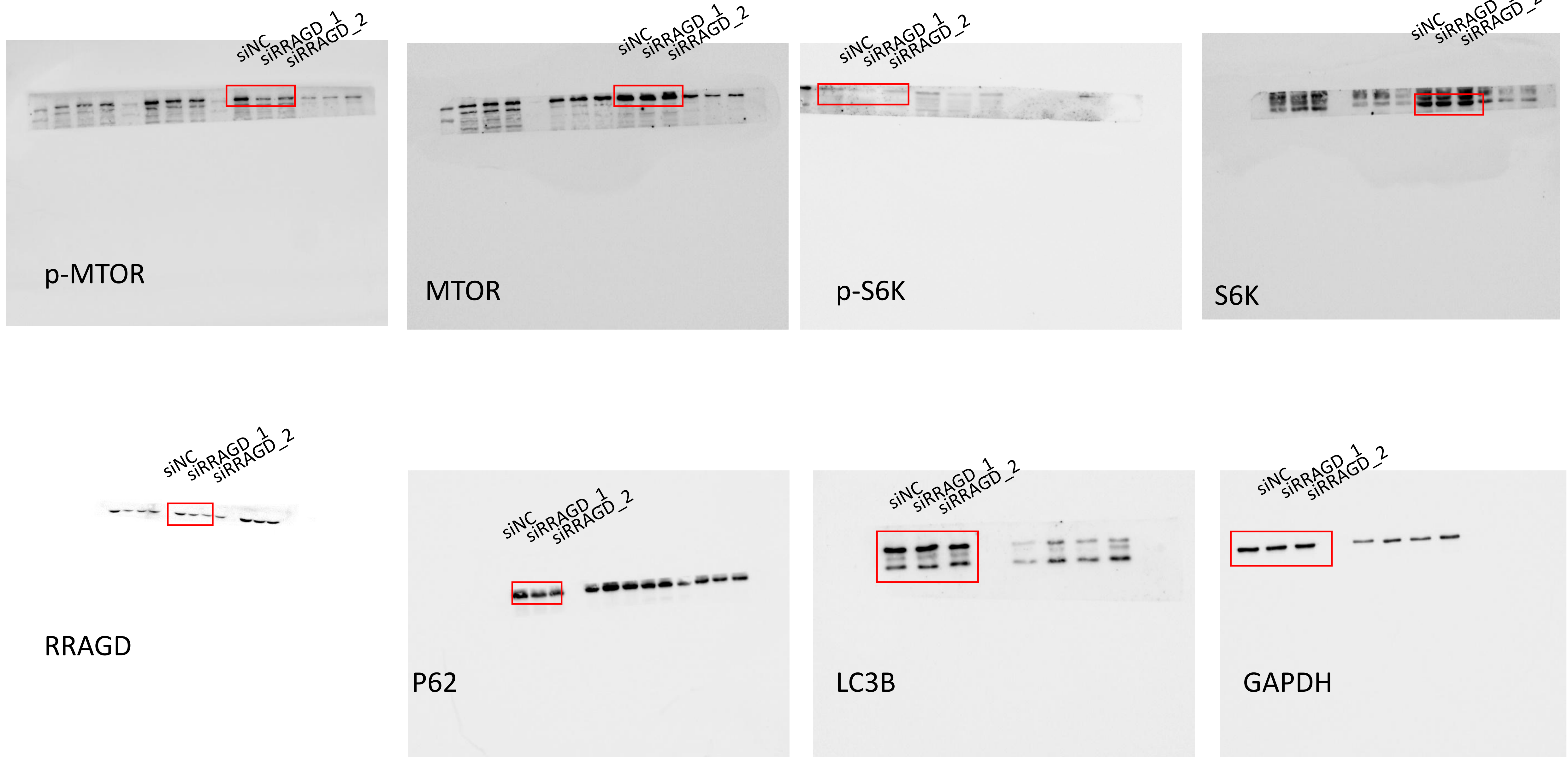
siNC
siRRAGD_1
siRRAGD_2
p-MTOR
MTOR
p-S6K
S6K
RRAGD
P62
LC3B
GAPDH

Fig.S7F

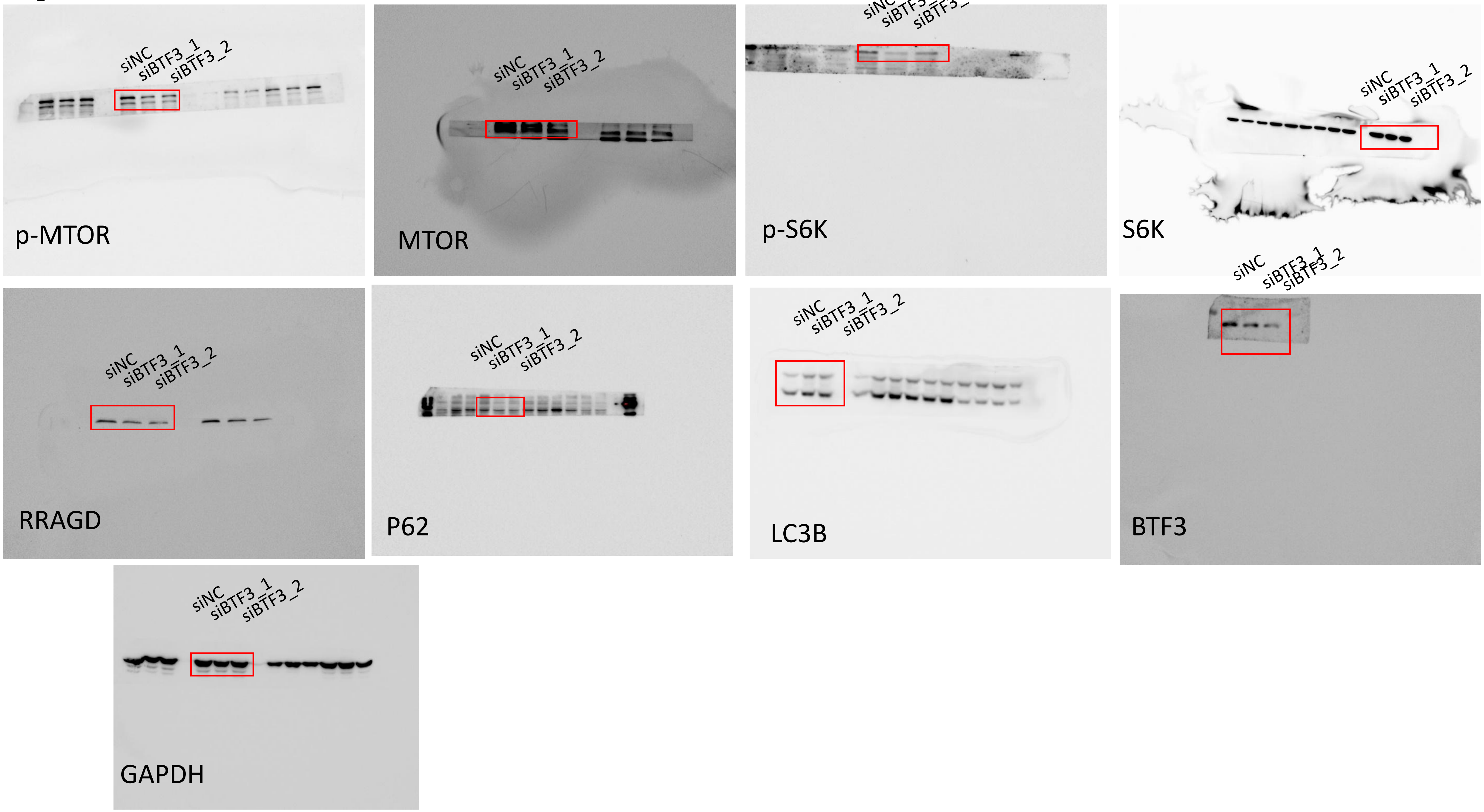

siNC
siBTF3_1
siBTF3_2
p-MTOR
siNC
siBTF3_1
siBTF3_2
MTOR
siNC
siBTF3_1
siBTF3_2
p-S6K
siNC
siBTF3_1
siBTF3_2
S6K
siNC
siBTF3_1
siBTF3_2
RRAGD
siNC
siBTF3_1
siBTF3_2
P62
siNC
siBTF3_1
siBTF3_2
LC3B
siNC
siBTF3_1
siBTF3_2
BTF3
siNC
siBTF3_1
siBTF3_2
GAPDH

Fig.S7I

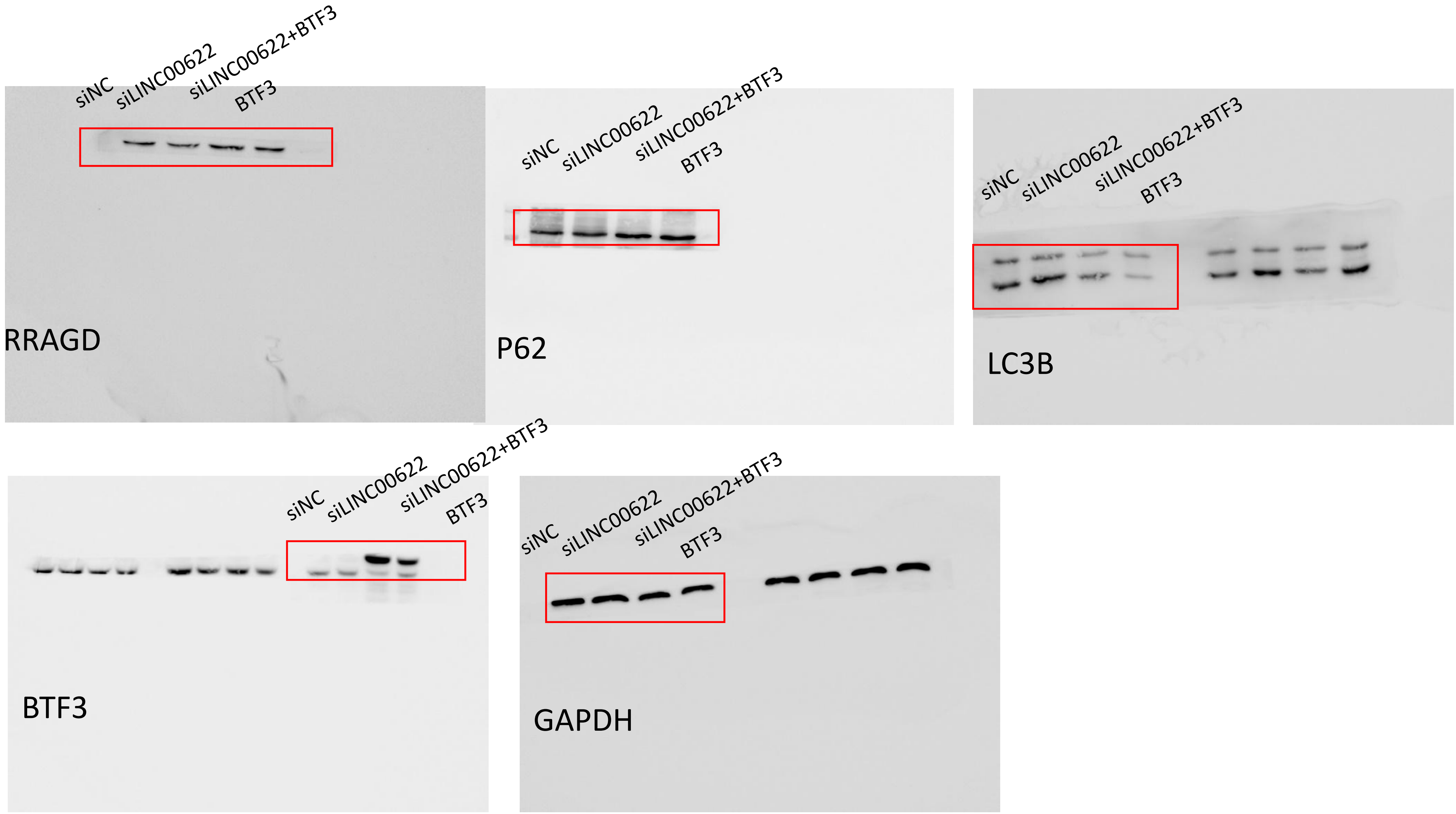
siNC
siLINC00622
siLINC00622+BTF3
BTF3
RRAGD
siNC
siLINC00622
siLINC00622+BTF3
BTF3
P62
siNC
siLINC00622
siLINC00622+BTF3
BTF3
LC3B
siNC
siLINC00622
siLINC00622+BTF3
BTF3
BTF3
siNC
siLINC00622
siLINC00622+BTF3
BTF3
GAPDH
